# Testing the effects of the Shamiri Intervention and its components on anxiety, depression, wellbeing, and academic functioning in Kenyan adolescents: study protocol for a five-arm randomized controlled trial

**DOI:** 10.1186/s13063-021-05736-1

**Published:** 2021-11-22

**Authors:** Katherine E. Venturo-Conerly, Tom L. Osborn, Akash R. Wasil, Huong Le, Emily Corrigan, Christine Wasanga, John R. Weisz

**Affiliations:** 1 Shamiri Institute, Nairobi, Kenya; 2 Department of Psychology, Harvard University, Cambridge, MA USA; 3 Shamiri Institute, Allston, MA USA; 4 Department of Psychology, University of Pennsylvania, Philadelphia, PA USA; 5 Department of Psychology, Kenyatta University, Nairobi, Kenya

**Keywords:** Adolescents, sub-Saharan Africa, Global Mental Health, Depression, Anxiety, Wellbeing, Shamiri

## Abstract

**Background:**

Treatments for youth mental disorders are a public health priority, especially in sub-Saharan Africa (SSA), where treatment options remain limited due to high cost, elevated stigma, and lack of trained mental health professionals. Brief, accessible, and non-stigmatizing community-based interventions delivered by lay providers may help address treatment needs in SSA. One such intervention, the Shamiri Intervention, consisting of three elements (growth mindset, gratitude, and value affirmation) has been tested in randomized controlled trials with school-going Kenyan adolescents. This three-element Shamiri Intervention has been shown to significantly reduce depression and anxiety symptoms and improve social support and academic performance relative to a control group. In this trial, we aim to investigate the effects of each element of the Shamiri Intervention.

**Methods:**

In this five-arm randomized controlled trial, we will test each of the intervention components (growth mindset, gratitude, and value affirmation) against the full Shamiri Intervention and against a study skills control intervention. Students (*N*_planned_ = 1288) at participating secondary schools who are interested in participating in this universal intervention will be randomized in equal numbers into the five groups. The students will meet in groups of 8–15 students led by local high school graduate lay providers. These lay providers will receive a brief training, plus expert supervision once a week throughout the intervention delivery. Multi-level models will be used to compare trajectories over time of the primary outcomes (depressive symptoms, anxiety symptoms, academic performance, and wellness) and secondary outcomes in each intervention group to the control group. Multi-level models will also be used to compare trajectories over time of the primary outcomes (depressive symptoms, anxiety symptoms, academic performance, and wellness) and secondary outcomes of participants in the single-element interventions compared to the full Shamiri Intervention. Finally, effect sizes (calculated as mean gain scores) will be used to compare all groups on all measures.

**Discussion:**

This trial will shed light on the mechanisms and outcomes targeted by each individual intervention, helping prioritize which mental health interventions are most important to disseminate.

**Trial registration:**

PACTR Trial ID: PACTR202104716135752. Approved on 4/19/2021.

**Supplementary Information:**

The online version contains supplementary material available at 10.1186/s13063-021-05736-1.

## Administrative information

Note: the numbers in curly brackets in this protocol refer to SPIRIT checklist item numbers. The order of the items has been modified to group similar items (see http://www.equator-network.org/reporting-guidelines/spirit-2013-statement-defining-standard-protocol-items-for-clinical-trials/).

**Table:** 

Title {1}	Dismantling the Shamiri Intervention for Anxiety, Depression, Wellbeing, and Academic Functioning in Kenyan Adolescents: Study Protocol for a Five-Arm Randomized Controlled Trial
Trial registration {2a and 2b}.	Pan African Clinical Trials Registry: PACTR202104716135752
Protocol version {3}	1.0
Funding {4}	All funding for this trial was provided by the Templeton World Charity Foundation, grant # TWCF0509
Author details {5a}	^1^Shamiri Institute, Nairobi, Kenya & Allston, MA, USA ^2^Department of Psychology, Harvard University, Cambridge, MA, USA ^3^Department of Psychology, University of Pennsylvania, Philadelphia, PA, USA ^4^ Department of Psychology, Kenyatta University, Nairobi, Kenya
Name and contact information for the trial sponsor {5b}	Templeton World Charity Foundation. Phone: 2423624904
Role of sponsor {5c}	This study was funded by the Templeton World Charity Foundation, located at Bayside Executive Park, Bldg. #2, West Bay Street and Blake Rd., PO Box N-7776, Nassau, Bahamas. The authors, and not the funding agency, are responsible for study design; collection, management, analysis, and interpretation of data; writing of the report; and the decision to submit the report for publication.

## Introduction

### Background and rationale {6a}

Many epidemiological studies have shown that common mental health problems are prevalent among adolescents aged 13-to-19 in low-income regions of Sub-Saharan Africa [1–3]. Unfortunately, these youths often cannot access treatment because of barriers such as societal stigma [4], lack of culturally appropriate treatment options [5], the length and cost of traditional treatments [6–8], and a paucity of trained mental health care providers [9]. Because a majority of mental health problems first arise during adolescence and have been shown to impact important future life outcomes [6, 10, 11], there is an urgent need for mental health interventions for youths in SSA where nearly half of the population is aged 19 or younger [12, 13].

Numerous youth interventions have been tested and found to produce beneficial effects [14, 15]. However, most of these interventions focus explicitly on psychopathology and thus could invoke stigma in SSA youths, and most require implementation by professionally trained providers, which are scarce in SSA countries. An alternative approach that may be more appropriate for SSA youths can be found in the emerging science of “character strength” interventions. Interventions that target character strengths (e.g., gratitude, sense of purpose) may circumvent the stigma associated with interventions that target maladaptive cognitions, behaviors, and emotions. Character strength interventions are commonly evaluated as positive psychology interventions [16] as well as “wise” interventions [17] that focus on a specific skill or concept [17–19]. Indeed, a body of research suggests that character strengths interventions may improve adolescent health, development, and academic outcomes. Character strength interventions may be particularly valuable in SSA and other high-stigma, low-resource settings because they are simple and likely low-stigma, meaning that they can overcome limitations including the paucity of mental health experts and societal stigma.

Building on the promising research that has supported the effects of character strength interventions on adolescent mental-health outcomes, our team has focused on developing and testing a simple, scalable, low-stigma, and low-cost intervention called Shamiri (Kiswahili for “thrive”) for Kenyan adolescents [20–22]. The Shamiri intervention includes three empirically supported brief character strength interventions: growth mindset [23–27], gratitude [28–30], and value affirmation [31, 32], which were iteratively adapted through co-design with and feedback from local stakeholders [20, 33] to be appropriate for use in Kenyan high schools. Shamiri is a group-based intervention delivered in naturalistic school settings by lay providers who are recent high school graduates without formal training in mental health [20, 21]. We tested the Shamiri intervention in a randomized controlled trial (RCT) with 51 Kenyan adolescents with clinically elevated depression and/or anxiety symptoms; youths assigned to Shamiri reported significant improvements in depression and anxiety symptoms, academic performance, and perceived social support from friends when compared to youths in an active study skills control group of equal duration [16]. In a well-powered replication (*N* = 413) [21], we found that youths assigned to the Shamiri intervention reported significantly greater improvement in depression and anxiety symptoms than youth in the control group. Effects lasted until the 7-month follow-up period [33]. Besides this, both RCTs showed that the Shamiri intervention was feasible and acceptable for Kenyan youths.

This past work may raise questions about whether the promising effects of the Shamiri intervention are due to the “bundling” together of three-character strength interventions, or whether some character strength interventions are more helpful than others, or even potentially beneficial when used alone. Answering these questions may shed light on the mechanisms and outcomes targeted by each individual intervention, as well as helping prioritize which mental health interventions are most important to disseminate. Previously, we investigated the effects of each element of the Shamiri intervention in a universal, classroom-based microtrial with 2-week follow-up [34]. This trial provided some initial evidence that the value affirmation and growth mindset components may be more effective on their own than the gratitude component, particularly for reducing symptoms of anxiety [34]. However, this trial had several limitations: first, it did not allow for comparison between the individual elements and the three-component Shamiri; second, the trial was cluster-randomized, and therefore underpowered to detect small effects; and third, microtrials are not intended to assess long-term effects. Thus, to build on our existing work, we plan to conduct a large, well-powered randomized controlled trial to compare the longer-term follow-up effects of the combined Shamiri intervention and its constituent character strength interventions (i.e., growth mindset, gratitude, and values), against an active control group.

### Objectives {7}

The objectives of this study are threefold. First, we aim to compare the effects of each intervention and control condition on the primary outcomes (depressive symptoms, anxiety symptoms, academic performance, and wellness). We hypothesize that all the intervention groups will outperform the active control on each primary outcome. Second, we aim to compare the effects of each group on secondary outcomes (described below in {12}). Finally, in an exploratory fashion, we also aim to identify for whom these interventions are most effective and their mechanisms of change.

### Trial design {8}

This study is a parallel-group randomized controlled comparative effectiveness trial with five arms:1) the Combined Shamiri Intervention (consisting of a growth element, a gratitude element, and a value affirmation element; henceforth called “Shamiri”), 2) the Growth Intervention, which encompasses content related to growth mindset and strategies for growth, 3) the Gratitude Intervention, which incorporates content to build feelings of gratitude and expression of gratitude, 4) the value affirmation intervention, which incorporates content to help students identify and better act on their personal values and sense of purpose, and 5) a study skills control group, an active control group teaching students study skills that may be useful to them as students. Each condition is described further below under “interventions.” Participants will be allocated within each school to the five conditions with a 1:1:1:1:1 ratio, or equal probability for assignment to each condition. Each arm will require 4-h-long sessions spanning 4 weeks and will be delivered in a group (8–15 students) setting by a local, trained lay provider between the ages of 18 and 24. Measures of mental health, wellbeing, and character strengths will be collected at baseline, 2-week midpoint, 4-week endpoint, and 1-month, 3-month, 6-month, and 9-month follow-up. Academic data will be collected for the school terms before, during, and after the intervention. De-identified trial data will be available in an online repository and upon request.

## Methods: participants, interventions, and outcomes

### Study setting {9}

The study will take place in high schools (i.e., secondary schools); we will seek up to 12 schools in the Nairobi, Kiambu, Machakos, Makueni, and neighboring counties in Kenya, to fill the target sample of 1288 students (see “Sample size” section below). Care will be taken to select a variety of schools: day and boarding schools, boys and girls schools, and, if possible, mixed-gender schools, and schools with a variety of rankings and incomes (for example, some schools ranked as “National” schools and others ranked as “County” or “Sub-County” schools; see [3] for more). A full list of study sites will be available on PACTR: https://pactr.samrc.ac.za/TrialDisplay.aspx?TrialID = 14677.

### Eligibility criteria {10}

Youths will be eligible for inclusion if they are a student in a participating school between the ages of 12 and 21. This will be a universal intervention and participants will not be screened in any way in, so long as they are in Form 2 and Form 3 (second and third years in high school). Participants who fail attention checks during baseline measures will be excluded. No participant who meets these criteria will be excluded for any other reasons other than the capacity of the groups: should more students than we can have in the group be interested in participation, we will randomly select students to join the groups.

The lay providers delivering the intervention will be eligible if they are living near Nairobi, Kenya, if they attended and graduated from a Kenyan high school, and if they are between the ages of 18 and 24. Among those applicants who meet criteria, the study team will conduct interviews to select those who seem to have the strongest counseling skills, commitment to the project, and understanding of the challenges commonly faced by Kenyan high schoolers. Applicants will first be evaluated via a brief written application, then via 30-min interviews including questions about experiences, motivations, and hypothetical scenarios. Full information about the process of interviewing and evaluating lay provider applicants can be found in [35]. Additionally, the study team will prioritize hiring those between 18 and 22. The recruitment and training process for lay providers in this trial will closely resemble that employed in a previous RCT of the Shamiri Intervention [35].

### Who will take informed consent? {26a}

A member of the study team who is from and living in Kenya (either Co-PI Wasanga or Co-I Osborn) will seek informed consent and assent prior to participant randomization and responses to baseline measures. We will provide all participants with a paper copy of the consent form (see {32} for information about a model consent form). Then, an upper-level member of the study team with appropriate ethical training and IRB approval will walk the participants through the consent form and clarify any questions that they may have. They will emphasize the voluntary nature of participation in the study.

### Additional consent provisions for collection and use of participant data and biological specimens {26b}

N/A; this trial does not involve collection or analysis of biological specimens.

## Interventions

### Explanation for the choice of comparators {6b}

The study skills control intervention, like the other active interventions described below, lasts for 4 h spread across 4 weeks. It is an active control intervention designed to control for all the non-specific aspects of group psychotherapy, including meeting in a group of students with a trained lay provider once a week, having discussions, completing activities in-session, and completing homework assignments. We selected this control condition for several reasons: 1) meta-analyses support the fact that active control conditions provide a more rigorous standard of comparison than waitlist controls [15], and 2) because students in Kenya suffer at high rates from undue family, school, and peer-induced pressure to succeed academically [36], there is good reason to believe that this study skills control intervention will somewhat alleviate symptoms of mental health problems and generally be useful to students; previous trials using a study skills control arm in this population support the fact that it has some effects on mental health problems [20], and 3) having an active control condition allows students regular contact with members of the study team, such that if any student is experiencing serious psychological concerns or is in danger, the barrier would be relatively low for them to approach their group leader with those concerns.

### Intervention description {11a}

#### Growth intervention

The growth mindset intervention consists of four small group (8–15 students) sessions delivered by Kenyan lay providers, lasting 1 h each. The intervention challenges the assumption that personality traits and intelligence are fixed. It is designed to strengthen individuals’ belief that the brain can adaptively respond to obstacles in various aspects of life (e.g., academic, interpersonal, and personality traits). Sessions will also focus on teaching strategies for growth such as problem solving, and each session will be followed by a homework assignment for further practice and consolidation. Previous growth mindset interventions have been found to enhance school performance, lower stress, and reduce symptoms of mental health problems in adolescents [23, 25, 34].

The intervention includes didactics, activities, and group discussion. During session 1, participants first learn about growth mindset and about research on neuroplasticity showing that our brain is able to adapt and grow as we act and think differently, thus enabling us to improve and grow over time with effort. Then, in session 2, they read and discuss testimonials depicting others’ experiences with growth mindset, write their own growth stories, and participate in a group discussion led by the lay provider to share these stories. They also complete a “saying is believing” activity in which they write a letter to a friend about what they have learned. In session 3, the trained lay provider leads activities and discussion about effective strategies for growth, including problem solving. In the final session, the groups then practice what they have learned by setting specific goals for growth and considering how they might achieve them, before reviewing everything they have learned and considering which parts may be most useful to them in the future. The full growth mindset protocol can be found in supplementary materials.

#### Gratitude intervention

The gratitude-only intervention focuses on promoting intentionally noticing, communicating, and appreciating feelings of thankfulness. Throughout the four 1-h small group (8–15 students) sessions, participants learn the concept of gratitude and apply the skills necessary for practicing gratitude in their interpersonal relations, academics, and other aspects of life. Meta-analyses have demonstrated that gratitude interventions can result in decreases in depressive symptoms and have benefits for wellbeing, mood, happiness, and life satisfaction [28, 29]. Additionally, previous trials have shown positive relations between gratitude and wellbeing in early adolescence [30, 37] and late adolescence [38].

In the gratitude intervention sessions, participants first learn didactic information about gratitude and its effects on our wellbeing and discuss their understanding of and experiences with gratitude in their own lives. In session 2, they hear testimonials from others for whom gratitude and appreciative behaviors improved their lives. They then write about and discuss their experiences with gratitude and how they can best go about expressing gratitude. Then, through subsequent in-session activities in session 3, they consider things about themselves for which they can be grateful, consider and practice new ways of expressing their gratitude, and write a gratitude letter to someone they especially want to thank. In the final session, they practice savoring good experiences in the moment and have a concluding discussion to review all they learned and consider which aspects of it they will use in their life going forward. As in the other conditions, participants also receive take-home assignments each week (such as an activity in which they notice three good things a day) to implement what they have learned and start forming a habit of giving thanks. The full gratitude-only protocol can be found in supplementary materials.

#### Value affirmation intervention

The value affirmation intervention, lasting for four 1-h sessions in a small group of 8–15 students, aims to teach the concept of noticing and living according to personal core values, thus contributing to a sense of purpose. Participants practice self-reflection on specific values that are most important to them, learn the necessary skills to apply values to their life, and set goals that are aligned with their values. Value affirmation interventions have been shown to help improve grades and to help support the sense of personal worth and integrity [31]. Past clinical trials that used value-based interventions reported reduced severity of depression symptoms in patients [39]. Additionally, results from our recently published study on Kenyan adolescents, in which this value-based approach—a component of the Shamiri intervention—was used, suggest that promoting value reflection may reduce mental health problems and improve psychosocial outcomes in adolescents in sub-Saharan Africa [20, 34].

This value affirmation intervention opens with the lay providers introducing and leading a discussion about the concept of values. The session includes activities and discussions covering topics such as examples of possible values, the positive effects of acting in accordance with your values, and examples of times when values might be relevant in life (e.g., when you are facing a challenge in class or trying to cope with a bad day). Students then discuss in small groups their own understanding of values, as well as some of the most relevant values to them. In session 2, participants are presented with testimonials of culturally pertinent role models with a focus on the values they demonstrate, and how these values have helped lead these individuals to live happy and successful lives. During this session, participants receive a sheet with a list of values and select some of the relevant values for a writing activity, which asks participants to reflect on times when they demonstrated these important personal values. Participants are asked to reflect on how they can live more in accordance with their values. In session 3, they learn to set short- and long-term goals related to their values and plan how to accomplish them. Through a letter-writing activity, they are asked to write a letter to someone that demonstrates values they personally admire. Session 4 includes thinking through how values could inform actions in a series of hypothetical, and then real, scenarios, during which students apply their values to come up with paths forward to handle problems. Finally, the participants have a concluding discussion to review what they learned and consider what they will use in the future. As in the other conditions, participants receive weekly homework assignments to promote use of the concepts learned in sessions to their daily lives. The full Value Affirmations protocol can be found in supplementary materials.

#### Shamiri intervention

The Shamiri Intervention, like the other interventions above and the control condition below, consists of four small group (8–15 students) sessions lasting for 4 weeks. The first two sessions focus on the concept of growth mindset and strategies for growth, the third session focuses on gratitude, and the fourth and final session focuses on value affirmation. Additionally, participants receive between-session homework assignments to help reinforce what they have learned and integrate the new skills into their lives. The Shamiri intervention is based on the three evidence-based single-component interventions above, termed “wise interventions,” that have been demonstrated to improve youth mental health outcomes [17, 18, 20]. Wise interventions may address mental health problems more efficiently in low-resource, high-stigma regions as they focus on overall human functioning instead of mental disorders, are conducted in a brief period of time, and require minimal training of lay providers. When previously trialed with Kenyan youths, the Shamiri intervention has been shown to improve not only youths’ depression and anxiety symptoms, but also their academic performance and perceived social connections with friends [20].

During session 1, students are guided through a growth mindset approach and how it allows individuals to thrive amidst life challenges. They learn the didactic information about “growth” and “neuroplasticity” and hear testimonials related to individuals’ academic/professional, interpersonal, and emotional growth. In session 2, participants are introduced to effective strategies for growth such as problem solving. They complete a saying is believing activity by writing a letter advising a hypothetical high school friend about a life challenge to help them understand and apply what they have learned. In session 3, participants learn the concept of gratitude and its benefits for wellbeing. They subsequently participate in a series of activities to apply this concept. One activity includes writing a gratitude letter to someone they wish to thank, and another asks participants to consider things about themselves for which they are grateful, while another asks participants to list three good things they are grateful for each day. In the fourth and last session, participants are introduced to the concept of values. They discuss stories of culturally pertinent role models, the values these individuals display, and how these values allow these individuals to lead successful and happy lives. Students also complete a writing activity about times when they have demonstrated one core value they select from a provided list and consider specific ways in which they could act more in accordance with their values in the future. The full Combined Shamiri protocol can be found in the supplementary materials.

#### Study skills control

The study skills control intervention, like the other active interventions, lasts for 4 h spread across 4 weeks. It is an active control intervention designed to control for all the non-specific aspects of group psychotherapy including meeting in a group of students with a trained lay provider once a week, having discussions, completing activities in-session, and completing homework assignments. Additionally, because students in Kenya experience high rates of depression and anxiety symptoms from undue family, school, and peer-induced pressure to succeed academically [3, 40], there is good reason to believe that this study skills control intervention will somewhat alleviate symptoms of mental health problems. Previous trials using a study skills control arm in this population support the fact that it has some effects on mental health problems [20].

This study skills control intervention includes exercises and group discussions about study strategies to improve academic performance, with a particular focus on note taking and an effective cycle of studying activities (e.g., previewing content, attending class, reviewing content, conducting self-assessment). Within-session activities include reading a short article to practice note taking strategies, sharing in pairs their understanding of the strategies discussed, and discussing the study strategies presented and their applications as a large group. Between-session take-home assignments include filling out worksheets that enable participants to further practice the study skills and reflecting on how their academic experience has changed as a result of the skills. The full Study Skills protocol can be found in supplementary materials.

### Criteria for discontinuing or modifying allocated interventions {11b}

We may discontinue allocated interventions in the case that a participant decides to withdraw from the intervention to which they were assigned, if a participant is highly disruptive during the groups and refuses to modify their behavior, or if a study PI deems it necessary for a participant’s safety and wellbeing to withdraw from the study. We anticipate that some students may choose to leave the study, but that few to no students will need to be removed from the study. We have no plans to modify allocated interventions once the trial begins, though prior to implementing the groups, it is possible that the interventions might be tweaked slightly in response to feedback from trainees or other stakeholders. Final intervention protocols will be published along with manuscripts and on OSF (https://osf.io/vynw9/).

### Strategies to improve adherence to interventions {11c}

The study team will employ three strategies to promote and measure adherence to intervention procedures. The first strategy is a thorough training, in which all group leaders will be trained didactically and through role-plays [32, 41] to deliver each intervention and control condition, to use the emergency protocol when necessary, and to adhere to general rules of trial conduct such as keeping participant information confidential and respecting professional boundaries. This training procedure will be modeled after that used in a previous trial of Shamiri [35], though modified and lengthened to suit the five-group study design.

The second strategy to promote adherence is supervision procedures, which will include weekly in-person supervision meetings led by trained supervisors to review intervention content, and daily supervision to ensure adherence to the emergency protocol and intervention protocols. All supervisors will be Kenyans with at least a BA in psychology and prior experience counseling. All supervisors will also receive at least 2 weeks of formal training in ethical trial conduct, trial procedures including emergency procedures, and the interventions being tested. Finally, these supervisors will be supervised by a PhD-level member of the study team (CW). The final strategy is to measure adherence to the protocols by asking independent raters to assess a randomly selected sample of session recordings (overall making up 10% of intervention sessions) for fidelity and performance, in a manner modeled after previous trials [20]. Each session will be rated by two independent raters, and their agreement will be calculated using Gwet’s AC2 for ordinal rating scales [42].

### Relevant concomitant care permitted or prohibited during the trial {11d}

Participants will not be prohibited from seeking other forms of care or using medication during this trial; however, because there are very few mental health providers [9] and elevated stigma in Kenya [4], it is unlikely that participants will seek professional mental health care outside of the trial. Additionally, as discussed under {22}, participants referred to the local PI for a risk assessment who present with significant risk of harm to self or others will be strongly encouraged to seek professional mental health services during the trial with a local mental health professional who is an affiliate of the study team. If we determine that a student needs continued post-trial care, we will coordinate that with the school administrators. We do not plan to remove the students who are referred to outside care from the sample unless the PI determines that they should be removed from the groups for their wellbeing.

### Provisions for post-trial care {30}

As discussed further under {22}, during the trial, participants who present to the study team with potential risk of harm to self or others will be assessed and re-assessed periodically for level of risk according to the emergency protocol (see supplementary materials for full emergency protocol), and, when the study PIs deem it necessary, these students will be strongly encouraged to seek individual psychotherapy with local clinicians affiliated with the study team during and/or after the trial. We do not anticipate that individuals will suffer harm because of trial participation and therefore have no plans for compensation for harm.

### Outcomes {12}

For full information on planned analyses, see section {20} below. In addition to statistical analyses described under {20}, means and standard deviations for scores on each outcome measure at each timepoint will be analyzed and reported.

#### Primary outcome measures

##### Patient Health Questionnaire-8

We will use the Patient Health Questionnaire-8 (PHQ-8) to assess the severity of youth depression symptoms. PHQ-8 is an 8-version item of the PHQ-9 that excludes the one item asking about suicidal ideation. We were advised by local researchers and school officials to exclude this item because the stigma associated with suicidal ideation might negatively affect the participating and potential students [43, 44]. PHQ-8 scores are highly correlated with PHQ-9 scores. Therefore, the same cutoffs can be used to assess depression severity [43]**.** The PHQ has been used in previous studies with both Kenyan adolescents [3] and adults [45, 46].

##### Generalized Anxiety Disorder Screener-7

We will use the Generalized Anxiety Disorder Screener (GAD-7) to assess levels of anxiety symptoms in the study participants. The GAD-7, which is used globally to detect anxiety among adolescent and adult populations [47], has been used in past studies of Kenyan adolescents and adults [3, 45].

##### Academic performance (average term grades)

Academic grades of the participants will be collected during (1) the school-term before the intervention (January to April 2021), (2) the school-term during the intervention (May to August 2021), and (3) the school-term after the intervention (September to November 2021). We will calculate the students’ average term grade (mean grade for each student across all enrolled subjects) to determine their academic performance per semester. Although the number of subjects per student differs across schools, students typically sign up for between 6 and 12 subjects. To compare students’ grades across different grade levels, academic subjects, and schools, we will use the standardization piloted in our pilot RCT to convert the academic grades to standard scores (M = 60, SD = 10, chosen arbitrarily and used in rescaling) [20]. We may also examine subject-specific scores in the same fashion, or Math and Science averaged scores separately from Humanities and Language averaged scores.

##### Short Warwick–Edinburgh Mental Wellbeing Scale (SWEMWBS)

The Short Warwick–Edinburgh Mental Wellbeing Scale (SWEMWBS) will be used to assess subjective wellbeing of the participants [48]. The SWEMWBS (7-item) is a short version of the Warwick–Edinburgh Mental Wellbeing Scale (WEMWBS), a 14-item tool developed for public mental health monitoring and assessment, which encompasses the promotion of mental wellbeing, the prevention of mental illnesses, and recovery from mental illnesses [49, 50]. Due to its lower participant burden, the SWEMWBS has been used extensively in large-scale psychological surveys/interventions in the UK and in non-English-speaking countries to evaluate clinical/psychological interventions and to assess the epidemiology of mental wellbeing [51–54]. The SWEMWBS and WEMWBS have previously been used in studies with South African youths aged 10–17 years [55] and adults [56].

#### Secondary outcome measures

##### EPOCH Measure of Adolescent Wellbeing (Engagement, Perseverance, Optimism, Connectedness, Happiness)-20

The EPOCH Measure of Adolescent WellBeing assesses five positive psychological characteristics: Engagement, Perseverance, Optimism, Connectedness, and Happiness, that might foster wellbeing, physical health, and other positive health outcomes in adolescence and later in adulthood [57]. The EPOCH has been validated with adolescents in Australia and the USA. It has also recently been used with Kenyan adolescents in an RCT assessing the Shamiri digital intervention for adolescent depression, anxiety, and wellbeing [22], in which it showed adequate psychometric properties. In this study, we will use all subscales of the EPOCH.

##### Purpose in Life Scale-12

We will use a 12-item Purpose in Life scale (PILS-12) to assess the student’s perceived meaning and life purpose and how these constructs relate to their wellbeing [58]. High scores on the PILS-12 suggest that the person experiences greater meaning and purpose in life, which are associated with greater self-confidence, self-acceptance, and experiencing more acceptance in life. On the other hand, low PIL scores have been found to be associated with despair and hopelessness and drug abuse [59, 60]. The PILS has been widely used for research and screening purposes, and the scale has been demonstrated to have strong psychometric validity [61]. Although the PILS-12 has yet to be used with adolescents in Kenya and other sub-Saharan African countries to the best of our knowledge, it has previously demonstrated adequate internal consistency in adolescents [58] and individuals suffering from depressive symptoms [62].

##### Gratitude Questionnaire-6

The 6-item Gratitude Questionnaire (GQ-6) is a well-established instrument that measures grateful disposition—the tendency toward noticing and appreciating positives, valuable support, and actions from others in everyday life [63, 64]. The GQ-6 items focus on the emotional component of gratitude and specifically measure span, frequency, and intensity of gratitude [64]. The GQ-6 has been shown to have strong psychometric properties among North American youths. It has also been used in past RCTs with Kenyan adolescents [3, 21].

##### Implicit Personality Theory Questionnaire-3

The Implicit Personality Theory Questionnaire (IPTQ) is a screening instrument used to assess the implicit theories of personality participants have about whether people’s attributes are fixed or malleable [26, 65, 66]**.** The IPTQ asks participants to indicate on a scale of 1 (really disagree) to 6 (really agree) the extent to which they acknowledge three statements: “You have a certain personality, and it is something that you can’t do much about,” “Your personality is something about you that you can’t change very much,” and “Either you have a good personality or you don’t, and there is really very little you can do about it.” The IPTQ score (ranging from 0 to 18) will be used to predict whether the person has a stronger entity (fixed) theory of personality or stronger incremental (malleable) theory of personality. The IPTQ has shown acceptable internal consistency across several studies, including studies assessing anxiety and depression symptoms in adolescents [23, 67].

##### Multidimensional Scale of Perceived Social Support – 8

The Multidimensional Scale of Perceived Social Support (MSPSS) measures personal satisfaction with social support [68]. It consists of three subscales: the “friends” subscale (which measures support from friends), the “family” subscale (which measures support from family), and the significant others subscale (which measures support from significant others). The MSPSS has previously demonstrated good internal reliability in previous studies of behavioral and psychosocial adjustment among Kenyan adolescents [3]. We will use the “friends" and “family" subscales of the MSPSS.

##### Acceptability of Intervention Measure-4

The Acceptability of Intervention Measure (AIM) is used to determine the extent to which an intervention is perceived to be acceptable by its participants [69]. The AIM has four items rated on a 5-point scale; items are averaged for each scale to yield a total score, ranging from 1 to 5. The AIM has demonstrated strong psychometric properties, including validity, reliability, and responsiveness to change. This measure has been implemented in studies on Kenyan adolescents or those experiencing, or at risk of developing, mental health difficulties [70, 71].

##### Intervention Appropriateness Measure-4

We will administer the four-item Intervention Appropriateness Measure (IAM) to assess whether participants believe the intervention is appropriate [53]. Similar to AIM, IAM has four items rated on a 5-point scale, with items averaged for each scale to yield a total score that ranges from 1 to 5. IAM has also demonstrated strong psychometric properties and has been implemented in studies with Kenyan adolescents and those experiencing, or at risk of developing, mental health difficulties [70, 71].

##### Demographic Questionnaire

The demographic questionnaire asks participants to report their basic demographic information, including their age, year in school, gender, family financial status, religion, county of origin, location of home (e.g., rural, urban), motivation to participate, and the ethnic background (i.e., tribe) they belong to.

##### Program Feedback Scale

The measure includes questions for participants about the interventions they participated in (e.g., which sessions they thought were most helpful) and whether they experienced change on hypothesized mechanisms of change (e.g., if they noticed themselves becoming more aware of their core values and sense of purpose as a result of the intervention). The measure also seeks other suggestions from participants about how the program can be improved for future implementation.

##### Perceived Control Scale for Children

The Perceived Control Scale for Children (PCSC) [72] aims to assess children’s perceived control (i.e., the belief that they are capable of achieving things that they want (or avoiding things they do not) with effort. The original 24-item questionnaire includes subscales reflecting perceived control over academic, social, and behavioral outcomes. A 6-item version of the original scale, devised using factor analysis and graded response modeling, will be used in the study.

##### Secondary Control Scale for Children

The Secondary Control Scale for Children (SCSC) assesses perceptions of secondary control, or one’s perceived ability to adjust oneself to hardships in such a way as to control their subjective emotional impact [73]. A 6-item version of the original 20-item scale, devised using factor analysis and graded response modeling, will be used in the study.

##### Mechanisms of Change

We will also include items designed to measure the extent to which participants have changed their thoughts and actions based on the character strength interventions. For each construct (gratitude, growth, values, and study skills), we will ask participants to complete two items designed to assess the extent to which participants have intentionally thought about the character strength or acted based on the character strength over the past 2 weeks (the items can be found in the supplemental materials). These items are intended to help us understand if the interventions produce their intended effect, as well as to understand potential mechanisms of change of each intervention.

##### Qualitative Participant Feedback

As part of the Program Feedback Scale completed post-intervention, every participant will be asked to provide qualitative feedback on their assigned condition including their favorite aspect of the program, what they would change, and any other feedback or comments. Additionally, at the second follow-up point (3-month follow-up), the study team plans to convene focus groups of participants to discuss their feedback on the condition to which they were assigned and any changes in their feelings, thoughts, and behaviors since participating in the program.

##### Group Leader Feedback Questionnaire

The feedback questionnaire asks for feedback from group leaders about the training they have received. There are two anonymous feedback questionnaires that group leaders will fill out; one will be distributed immediately post-training, and the other after the leaders have completed the program. Each questionnaire asks the group leaders to indicate their satisfaction with the training, on a scale of 1 (the training is terrible and makes them feel extremely unprepared) to 7 (the training is fantastic and makes them feel well-prepared to lead the sessions); the second section asks these leaders to circle, from 1 (strongly disagree) to 5 (strongly agree), their thoughts on the quality of the training (e.g., whether the training is taught clearly) and if they become comfortable learning from the Shamiri team (e.g., whether the Shamiri team clearly conveys expectations and respect toward the group leaders during the training). The last section includes several short answer questions that ask these group leaders to give written feedback about the program and suggestions for improvement.

### Participant timeline {13}

**Fig. 1.**
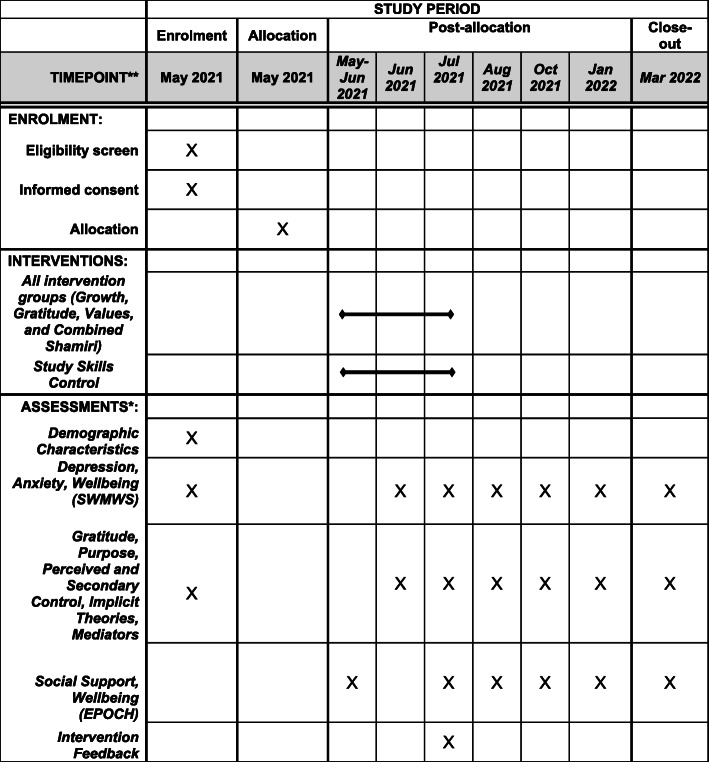
Schedule of enrollment, interventions, and assessments for the five-arm Shamiri intervention trial. *Note that academic outcomes will be collected as they become available, with the likely timeline being pre-intervention academic outcomes collected in May–June 2021, mid-intervention academic outcomes being collected in August–September 2021, and post-intervention academic outcomes being collected in December 2021–January 2022

School recruitment will begin in May 2021, with participant recruitment then beginning in late May of 2021, and the consenting process occurring in late May of 2021 immediately prior to baseline assessment. Baseline assessment of demographic variables, some of which will be accounted for in randomization, and of primary and some secondary outcomes will be completed immediately after consenting. Within the n1ext week, allocation will occur, and the first intervention sessions will begin in late May or early June. Immediately prior to those sessions, students will complete other baseline measures of remaining secondary outcomes. At the 2-week intervention midpoint in June, participants will complete midpoint measures of primary outcomes and potential intervention mediators (i.e., some secondary outcomes) immediately post-session. At the 4-week intervention endpoint in late June or early July, participants will complete all primary and secondary outcome measures and measures of intervention feedback. Finally, at 1-month follow-up (August), 3-month follow-up (October), 6-month follow-up (January), and 9-month follow-up (March), participants will again complete all measures of primary and secondary outcomes. See Fig. 1 for the SPIRIT diagram depicting the intervention, assessment, and enrollment timeline.

### Sample size {14}

We conducted a power analysis using Optimal Design, a statistical simulation software which can estimate sample size for randomized controlled trials assessed using multi-level (i.e., mixed effects) models [74]. In Optimal Design, we estimated the required sample size for a person-randomized trial with six-time repeated measures (baseline, midpoint, post, 1-month follow-up, 3-month follow-up, 6-month follow-up, and 9-month follow-up). Additionally, for each outcome measure, we have four contrasts of primary interest (each intervention condition compared to the control), thus, using a conservative *p* value correction, we would divide the typical alpha value of .05 by four and get a *p* value threshold for significance of .0125. Finally, we assumed that we are interested in detecting a minimum post-treatment effect size of .3. We selected this value for the following reasons: 1) it is slightly lower than the effect sizes found in most previous trials of Shamiri, which range from .32–.83 at the latest follow-up points [3, 20, 33, 34], which will allow us to detect potentially smaller effects of the individual component interventions, and to detect effects in a universal sample, and 2) a standardized effects size of .3, which is considered small to medium, remains clinically meaningful and important in our view, whereas a change smaller than that may not be as clinically meaningful. Given these parameters, we found that a sample of 515 students would be needed to conduct a two-group trial with a power of .80. So, for this five-group trial, we multiplied that by 2.5, to get a final target sample size of 1288 students. Because in a past trial with 7-month follow-up there was a high dropout rate during follow-up of up to 40% [33], we will initially invite 2147 students to the sessions, assuming that around 40% of them will drop out prior to the sessions or during the follow-up period.

### Recruitment {15}

#### School recruitment

Officials at local high schools in Nairobi and nearby areas will be approached by the researchers to participate in the study. The high schools will discuss study participation in a conversation guided by a flyer that will outline details about the study. From interested schools, we will select 5–12 high schools. The selection of the participating high schools will be done to ensure that there is a mix of boy schools and girl schools and a mix of national and county, and day and boarding schools. We will also take into consideration logistical considerations (i.e., if the group sessions in two selected schools can only occur at the same time, most likely only one of the two schools will be selected). Additionally, a small in-kind donation of materials worth no more than 200 USD will be provided to participating schools to thank them for the time and work they have invested in the study and contribute to student wellbeing.

#### Participant recruitment

Youth participants from up to 12 Kenyan high schools will be recruited by the Kenyan study team members (Co-PI Wasanga and Tom Osborn) from local high schools. The intervention will take place at these high schools during the school day. The exact time will be determined in consultation with the schools and will depend on the scheduling needs of each school. In all the participating schools, students attending high school will be notified about the study by the school headteachers and other administrators. The school administrators will then gather interested students in classrooms. Eligible students will be informed that participation is voluntary and that they can opt out at any time, as described in the Participant Flyer. They will also be provided with an opportunity to ask questions, after which they will also provide informed consent/assent. All interested students will then be asked to fill out a brief baseline questionnaire battery to assist with randomization. As compensation for participation, all study participants will receive notebooks and pens/pencils during the first intervention session and will receive an opportunity each week to raffle for small prizes such as t-shirts or water bottles each week.

## Assignment of interventions: allocation

### Sequence generation {16a}

After the baseline assessment, youths who opt to participate in the study will be randomly allocated within each school to one of the four intervention conditions or to the study skills control condition (with equal probability for assignment to each condition) using a computerized random number generator in R. Randomization will be stratified by reported age and gender in the case of mixed-gender schools, as well as by level of symptoms at baseline (i.e., GAD-7 and PHQ-8 scores).

### Concealment mechanism {16b}

All students at a given school who choose to enroll in the study will be randomized at the same time by an offsite researcher who is uninvolved in the study planning and implementation. There will therefore be no way for the study team to predict or influence which students will be assigned to which condition, to know the allocation sequence, or to know which students have been assigned to which conditions until the offsite researcher provides the team with a final, full list of student assignments.

### Implementation {16c}

An offsite researcher will generate the allocation sequence and assign participants to interventions using this allocation sequence. Then, researchers from the study team located in Kenya will randomly assign group leaders to lead certain groups, and will enroll students into these groups (in the appropriate condition assigned by the offsite researcher) at each school.

## Assignment of interventions: blinding

### Who will be blinded {17a}

Participants, lay providers, and study staff working with participants and lay providers cannot be blind to condition; however, lay providers and participants will not be informed of study hypotheses, and the study will be described as testing a series of interventions to understand their effects on wellbeing and academic performance. This should help conceal the control group.

### Procedure for unblinding if needed {17b}

Unblinding will only be necessary in the event of a safety concern; in these cases, only those involved in implementing the intervention on the ground (i.e., group leaders, clinical supervisors, and the expert clinician on the study team) will need to know the individual’s identity and group assignment. All others, including data analysts and those involved in entering data, will be kept blind, and we do not expect that unblinding will be necessary during the trial for these other members of the study team. Thus, we do not have unblinding procedures in mind for anyone except those involved in direct provision of emergency clinical services. Additionally, data collection procedures will ensure blindness in two ways: (a) no measure administrators will know what any student’s response was to any question, and (b) participants will be informed at the beginning of the assessments that their responses will be confidential in this way.

## Data collection and management

### Plans for assessment and collection of outcomes {18a}

As discussed under {12}, primary outcomes include participants’ severity of youth depression symptoms (as measured by the PHQ-8), levels of anxiety symptoms (GAD-7), academic performance (standardized scores representing students’ mean grades across enrolled subjects), and mental wellbeing (SWEMWBS). Secondary outcomes include participants’ character strengths related to wellbeing (per the EPOCH), perceived meaning and life purpose (PILS-12), gratitude (GQ-6), implicit theories of personality (IPTQ), use of intervention strategies, and personal satisfaction with social support (MSPSS). Measures of intervention acceptability and appropriateness among participants (AIM and IAM, respectively) will also be collected, along with basic demographic information (Demographic Questionnaire). The study team will also elicit program feedback from both study participants and group leaders. Study scales and questionnaires, as well as their reliability and validity, are further detailed under {12}.

Measures of demographic variables and primary outcomes will constitute one baseline data collection form, to be administered and collected by the study team immediately following the consenting/assenting process. Lay providers will then pass out baseline measures of secondary outcomes under supervision from study staff by asking participants to complete a separate questionnaire battery with primary outcomes immediately prior to the first intervention sessions. These same lay providers will administer, under the supervision of study staff, measures of primary outcomes and potential mediators (including some secondary outcomes) at the intervention midpoint, and all primary and secondary outcome measures and intervention feedback measures at the endpoint. Trained supervisors and the Kenyan study PI (Wasanga) and Co-I (Osborn) will ensure data confidentiality and adherence to data collection protocols among lay providers at each of these stages. Supervisors will also collect physical questionnaires immediately from the group leaders, which trained study team members will scan and upload according to the procedures described under {27}.

Kenyan study team members (Co-PI Wasanga and Co-I Osborn) will work with school officials to administer the 1-month, 3-month, 6-month, and 9-month follow-up data collection forms, which will include all primary and secondary outcome measures. We will access academic performance data for the three school terms before, during, and after the intervention through publicly accessible datasets provided to us by participating schools. Academic performance data will be standardized according to the process described under {12} and piloted in our initial RCT [20].

The study team will promote data quality by training lay providers and supervisors on adhering to intervention protocols and rules of trial conduct, including proper data collection techniques. Additionally, all those who interact with private participant data (e.g., those scanning and collecting questionnaires and working with unblinded datasets) will have up-to-date formal human subjects training certificates, either from their institution of affiliation or from the federal government. Enlisting independent raters to code randomly selected recordings of intervention sessions for lay provider performance and fidelity will further enhance data quality. See {11c} for more on the above. Finally, we will employ periodic data entry checks and will use range checks on all data, as described under {19}.

### Plans to promote participant retention and complete follow-up {18b}

Study team members will work with school officials to promote participant retention. School officials will help the study team remind participating students to attend intervention sessions at the scheduled times and attend follow-up data collection sessions. The study team will also conduct raffles of small prizes (e.g., water bottles and t-shirts) for participants during intervention sessions and provide a small gift to each school (in the form of an in-kind donation of materials including some to prevent the spread of COVID-19).

Participants who miss any intervention session(s), with the exception of the first which includes essential baseline data collection, will not be removed from the study nor barred from attending subsequent sessions. The study team will continue to collect measures completed by such participants during any sessions and follow-up points they attend; of course, participants can stop attending sessions at any time and for any reason. Because school grades are publicly accessible via participating schools, we will still collect school grades for any participant who discontinues or is removed from the intervention after the first session for any reason listed under {11b}, unless the participant explicitly withdraws consent to participate in the study, in which case their data will be removed from all analyses.

### Data management {19}

At each stage of the intervention and follow-ups, the study team in Kenya with the involvement of either school administrators or lay providers will administer paper questionnaires to study participants. Trained supervisors or other trained members of the study team (all of whom have completed research ethics courses online and with the study team in person) will collect questionnaires and immediately bring them back to a secure, locked room at the study headquarters. We will use a software called Papersurvey.io (which is fully compliant with the European Union’s General Data Protection Regulation) to automatically scan and upload physical questionnaires with a scanner stored in this same secure location. A study team member will periodically verify data automatically scanned into Papersurvey.io against physical questionnaire responses in order to ensure that no systematic data entry errors arise. In addition to random data checks, the study team will range check all data values in the full dataset. Full data storage, scanning, and uploading protocols to promote confidentiality for both paper and digital questionnaires are detailed under {27}.

### Confidentiality {27}

We will take several steps to maintain confidentiality, including keeping paper questionnaires in a private location, separating identifying information from sensitive participant data, and making only fully de-identified data public. Data in paper format will be stored in a private location in a locked room. Paper copies of study data will be associated with a participant ID number assigned by study staff. Exceptions to this rule include consent and assent forms, which will include participant signatures and will therefore be stored in a locked location separate from other study data.

As noted under {19}, we will use a software called Papersurvey.io (which is fully compliant with the European Union’s General Data Protection Regulation) to automatically scan and enter responses to paper questionnaires. Once these questionnaires have been entered using Papersurvey.io, the scanned questionnaires and databases will be deleted from the Papersurvey.io server and will be moved to a password-protected and encrypted study database.

All study data will be de-identified and stored in a password-protected and encrypted database only accessible to study staff. Study staff will have access to this database via confidential usernames and passwords. All questionnaires, copies of consent forms, and audio recordings of group sessions will be stored in this password-protected and encrypted database. Audio recordings that are not selected to be coded will be deleted, as will those selected to be coded at the conclusion of the study. Additionally, a list of participant names and corresponding ID numbers will be stored in a separate, password-protected folder of the study staff’s password-protected and encrypted electronic database. Any identifying features of participants will be removed from the aggregate data presented to those outside the study team (e.g., in posters or manuscripts). Lay providers will know participant names and the information participants choose to share with them but will not be informed about scores on other measures associated with these participants. They and other study staff will be trained in the importance of maintaining participant confidentiality.

### Plans for collection, laboratory evaluation, and storage of biological specimens for genetic or molecular analysis in this trial/future use {33}

N/A; this trial does not involve collection or analysis of biological specimens.

## Statistical methods

### Statistical methods for primary and secondary outcomes {20a}

Outcome measures will only be analyzed if they demonstrate internal consistency within the full sample at baseline, defined as an alpha value of .70 or above [75]. For those outcome measures that pass this threshold, all main analyses of primary and secondary outcomes will follow an intent-to-treat approach, meaning that all participants who consent, are randomized into the groups, and complete baseline measures will be included in all analyses (see 20c below for more about plans for handling missing data).

For all analyses of primary and secondary outcomes, we plan to use linear mixed effects models to model time × condition interaction with a random intercept for participant ID to allow for individual variation at baseline. We plan to control for age and sex in all models of primary and secondary outcomes. Age will be included because older adolescents report increased psychosocial stress and thus greater risk of mental illness [3]. Similarly, sex differences in anxiety and depression are widely documented in adolescents including Kenyan adolescents [3, 76]. Additionally, the authors will test more complicated random effects structures which reflect the hierarchical structure of the data, including random effects for groups within group leaders, crossed random effects for schools and group leaders, and for participants in groups. We will also test adding a random slope for participant ID and for school; however, these added random effects may result in overfitting, which would reduce the generalizability of our findings. If this is the case, we will select a more parsimonious model with a simpler random effects structure. These variables (group leader, school, and group) will also be tested as covariates rather than random effects in the model, and final model selection will be done taking into account both overfitting warnings and Akaike Information Criterion (AIC) [77].

Primary models will include all five groups, using the control group as the contrast group. Secondary models will include the four intervention conditions, using the full Shamiri intervention as the contrast group. Significant (*p* < .05 with a Holm-Bonferroni correction to account for four comparisons within each model) time × condition interactions will indicate that one condition produced significantly steeper improvements than another condition across the time included in the analyses. As described above and per recommendations outlined in past literature [78], the Holm-Bonferroni correction will be applied at a family-wise level (i.e., to account for multiple group comparisons on each outcome), rather than at a study-wise level.

Additionally, for all pairwise contrasts (including those comparing active interventions to each other) on all primary and secondary outcomes, effect sizes will be calculated as mean gain scores, to compare change within the various intervention and control groups.

### Interim analyses {21b}

No interim analyses will be conducted during the intervention sessions, which are only 4 weeks long. Additionally, we do not anticipate systematic harm to participants due to study participation, thus we do not have study stopping guidelines in place. For individual participants who clinical supervisors deem as having serious risk of harm to self or others, the local expert clinician could decide, in collaboration with the school administration, to withdraw the participant from the trial and instead provide more intensive individual support. See supplemental emergency protocol documents for more. However, the outcomes from 2-week midpoint, 4-week endpoint, and 1-month follow-up will most likely be analyzed prior to those at 3-month, 6-month, and 9-month follow-up, and will accordingly be reported separately. This is because of the large number of groups and outcomes, which would likely prohibit reporting all in one manuscript.

### Methods for additional analyses (e.g., subgroup analyses) {20b}

As part of our main analyses, we do not plan to carry our additional subgroup or adjusted analyses. However, we plan to conduct exploratory analyses such as moderator and mediator analyses and epidemiological analyses using the trial data. Such analyses will be published separately from the main analyses of primary and secondary trial outcomes.

### Methods in analysis to handle protocol non-adherence and any statistical methods to handle missing data {20c}

Missing item and subject-level data will be imputed at least five times using Fully Conditional Specification (FCS), implemented using the multivariate imputation by chained equations (mice) package in R [79]. Multi-level models will be built using the pooled imputed datasets. Additionally, as appropriate, we may report results using the un-imputed dataset or conduct other sensitivity analyses using different styles of data imputation or using last observation carried forward. As described further under Strategies to Improve Adherence to Interventions, a selection of session recordings will be rated by independent raters for fidelity, and results of these ratings will be reported. If the study team or independent raters identify systematic deviations from the protocol by certain lay providers, students who received counseling from these lay providers may be removed from the dataset for sensitivity analyses.

### Plans to give access to the full protocol, participant-level data, and statistical code {31c}

The authors plan to grant full public access to the trial protocols and training materials, to participant-level data upon request once it is fully de-identified and cleaned, and to statistical code for published analyses.

## Oversight and monitoring

### Composition of the coordinating center and trial steering committee {5d}

The coordinating center is located at the Shamiri Institute headquarters in Kiambu, Kenya (near Nairobi), a secure, locked building with secure, locked rooms inside of which the study team may safely store data. The trial steering committee consists of the study PIs (both PhD-level mental health clinicians), two doctoral students in clinical psychology, and an affiliate of Shamiri Institute with a bachelor’s in psychology. Approximately half of the trial steering committee is located in Kenya, while the other half is located in the USA. All members of the trial steering committee meet weekly to plan and manage the trial and discuss any issues that may arise; they will continue to meet weekly or bi-weekly when the study is implemented, and follow-up data collected. The trial steering committee oversees data management including collection and entry of data, plans day-to-day trial implementation and supervision for lay providers, oversees participant recruitment and consulting, and reviews and addresses safety events as they occur.

### Composition of the data monitoring committee, its role and reporting structure {21a}

The Data Monitoring Committee will meet with the study investigators once yearly to review trial progress and aggregate findings, as well as to review non-identifying reports of any adverse events and steps taken by the study team to address them. They will then provide their guidance about any further next steps that might help protect participant safety and wellbeing, and will opine as to whether adverse events were related to or independent from the trial. The Data Monitoring Committee may also be contacted outside of these yearly meetings if the investigators feel they would benefit from their input. The Data Monitoring Committee will be composed of several individuals who are uninvolved with the study but have expertise in youth mental health care in sub-Saharan Africa. Notably, these individuals will not receive any royalties from the Shamiri Institute or from the Templeton World Charity Foundation for their work. Thus, they will have no competing interests.

### Adverse event reporting and harms {22}

The emergency protocol for participant risk scenarios was developed using the American Academy of Pediatrics guidelines for child and adolescent suicide risk, which encourage explicitly asking about suicidal thoughts, intent, plans, and history, as well as assessing risk and protective factors and triangulating interview findings with symptom measures [80]. These guidelines also suggest employing sensitivity and having a natural conversation about how the youth is feeling, as opposed to simply firing off a series of questions about suicide risk [80]. Additionally, guidelines from the American Academy of Child and Adolescent Psychiatry suggest balancing participant confidentiality and comfort with the need to inform guardians (i.e., teachers in the case of this present study) of child or adolescent risk. In all cases in which harm may be imminent (i.e., high risk), it is necessary to inform the guardian of youth risk [80, 81]. However, in cases in which the harm is not intended or imminent, steps are less clear-cut and must involve balancing participant comfort and confidentiality with safety, where safety is most important [81]**.** In an effort to achieve the appropriate balance, we will inform school staff of students who study staff identify as currently at a medium or high risk of suicide; we will not routinely inform schools of low-risk cases, but our expert clinician will consider that step in low-risk cases if it seems appropriate. In all low-risk cases, study staff will continue monitoring and evaluation conversations weekly and inform the school if the student rises to a higher risk level. Those with medium risk or higher will be very strongly encouraged to seek professional help, facilitated by the study team and in coordination with guardians. Additionally, all cases of risk or other adverse events will be reported to the IRB of record, the KU IRB, and we will follow their guidance in addressing these events.

The study staff (including lay providers) will not systematically ask participants about risk in any questionnaires or in the group meetings. This is to avoid unnecessary stigma and discomfort. However, in the event that a student displays potentially serious mental health concerns (e.g., if a participant mentions thoughts of/desire to harm self or others) or other concerns that especially concern or overwhelm the lay provider, the lay providers will be trained to follow-up with the student privately and promptly (e.g., right after the group session) to further assess the situation—for example, by asking about thoughts of harm, and whatever other concerns the student has mentioned. Afterward, group leaders will be trained to promptly speak with their supervisor. The supervisor will then assist with triage (by asking about thoughts of harm, and, depending on the response, methods, plans, intent, and history). If appropriate and consistent with school policy, the supervisor will organize follow-up and referral to appropriate resources. All such conversations, even those that yield a designation of “no risk,” will be reported immediately to study staff, and all conversations that yield a risk designation of medium risk or higher will result in an immediate phone conversation with the local study PI, an experienced clinician. See emergency protocol in supplementary materials for more details.

If less urgent problems are uncovered in the risk assessment or elsewhere (e.g., academic troubles), we may, after consultation with the PIs and expert clinicians involved in the study, refer these students to the appropriate school administrator (e.g., school chaplain, school counselor, school principal). In these cases, we will follow the standard referral procedures used by these schools. For cases in which the study PIs disagree with each other or would like a third opinion about the need to consult the administration or about other potential steps, we will contact the Kenyatta University IRB to inquire about an appropriate path forward.

### Frequency and plans for auditing trial conduct {23}

For this trial, especially because the study procedures are very unlikely to have adverse effects on participants, we do not have plans for auditing apart from that described above done by the Data Monitoring Committee and any updates requested by the IRB of record or by the trial sponsor, the Templeton World Charity Foundation. If the investigators, sponsor, or IRB become concerned about any aspect of trial conduct, the study team will consider bringing in an independent auditor to help diagnose and resolve trial conduct issues.

### Plans for communicating important protocol amendments to relevant parties (e.g., trial participants, ethical committees) {25}

Any changes to trial protocols will be communicated first to the investigators through email, phone, or video conference in routine meetings, and then, when the investigators judge it appropriate, to the IRB of record (Kenyatta University IRB) via email, to the trial sponsor via email, and on the public, online PACTR trial registration page. When deemed necessary by the investigators or the IRB, trial participants will be notified about protocol changes via announcements at their schools.

### Dissemination plans {31a}

The authors plan to disseminate findings of this trial through manuscripts published in peer-reviewed journals, through data, manuscript, and code sharing on open access platforms such as OSF, and through presentations at academic conferences. Reporting of these results will be broken into several articles because of the large number of outcomes, groups, timepoints, and questions of interest in this study, and the limited word counts at almost all academic journals. Additionally, to the extent possible, the authors plan to disseminate intervention protocols and online versions of the intervention publicly online, and to publish results in popular media outlets. The authors will strive to make all published materials open access.

## Discussion

Here, we described the design of a comparative effectiveness randomized controlled trial designed to evaluate four school-based mental health promotion interventions delivered by lay counselors. Through this study, we will understand if the interventions produce an effect on depressive symptoms, anxiety symptoms, academic performance, and subjective wellbeing. We also aim to identify for whom these interventions are most effective, their mechanisms of change, and their potential impacts on secondary outcomes.

The results from this study will be used to inform future efforts (by our group and potentially others) to identify and disseminate the most effective, cost-effective, and culturally appropriate ways to promote mental health among Kenyan adolescents. This work will directly inform our efforts through the Shamiri Institute (https://www.shamiri.institute/), a non-profit organization that forms partnerships with community organizations in Kenya to implement evidence-based mental health and wellness interventions. The knowledge acquired from this trial (e.g., which character strength intervention(s) are most effective? For whom do these interventions have the strongest effect?) will directly inform our group’s efforts to continue to disseminate mental health interventions throughout Kenya. The knowledge and materials produced through this trial may also inspire and inform mental health promotion efforts in other LMICs, and even in high-income countries such as the USA, where it is estimated that only a third of youth in need of mental health care receive it [82].

The trial’s results should be interpreted in light of some scientific and practical limitations. First, we are testing these interventions as universal interventions, in which students are eligible to participate regardless of their level of symptoms. While there are advantages of this approach such as not requiring pre-screening and, likely, reducing stigma for participants, our findings may not generalize to targeted interventions, in which all group members meet a certain symptom threshold. Second, it is possible that training group leaders in the single-component interventions (gratitude, growth, and values) also improves their ability to perform the combined Shamiri intervention. While we will be able to measure group leaders’ fidelity to each of the interventions, it is possible that the nature of our trial (and training) could provide group leaders with more competence in delivering the combined intervention. Third, when performing studies in schools (and other naturalistic settings), researchers often have less control over the setting. As a result, there may be certain threats to internal validity (e.g., students sharing information about their group with other students, academic schedules requiring delays in the collection of data). In our view, these threats to internal validity are counterbalanced by benefits to external validity [83]. Furthermore, to promote the successful implementation of our study, our group will apply best practices we have learned through our previous work [84].

Future research will be needed to identify additional ways to promote mental health in LMICs. For example, future research could compare the effectiveness of character strength interventions (such as Shamiri) to traditional interventions like cognitive behavioral therapy. Previous studies have found that cognitive behavioral therapy and behavioral activation can be effective treatments for youths in LMICs [85, 86]. However, little is known about how these interventions compare to Shamiri or other character strength interventions. Another promising avenue for future research is the development and evaluation of remotely delivered mental health interventions, such as digital interventions, for youths in LMICs. Digital and other remote mental health interventions may be especially valuable in LMICs, given their widespread availability, low costs, and growing evidence base [87–91]. Although much of the evidence on digital mental health interventions for youths come from high-income countries, early efforts are already underway to adapt and evaluate online and smartphone-based self-help interventions in LMICs (e.g., [22, 92, 93]). Additionally, future research is needed to understand how to effectively develop and implement stepped-care systems in LMICs [94, 95]. Stepped-care systems may be especially useful in LMICs, where resources for mental health care are generally scarce and access to mental health care is highly limited. Such work could be guided by efforts to understand “what works for whom.” Understanding which youths benefit from low-intensity services, and which youths require higher-intensity services, could inform how stakeholders allocate individuals to different interventions.

Ultimately, a variety of efforts will be needed to expand access to evidence-based mental health care worldwide. It is our hope that our trial will be one of many efforts toward promoting youth mental health care worldwide.

## Trial status

Protocol Version 1.0 (5/07/2021)

Recruitment has not yet started, and we anticipate completion of recruitment on approximately June 1st, 2021.

## Supplementary Information

**Additional file 1.** .

**Additional file 2.** .

**Additional file 3.** .

**Additional file 4.** .

**Additional file 5.** .

**Additional file 6.** .

**Additional file 7.** .

**Additional file 8.** .

## Abbreviations

CBT: Cognitive behavioral therapy
SSA: Sub-Saharan Africa
RCT: Randomized controlled trial
PI: Principal investigator
PHQ-8: 8-item Patient Health Questionnaire
GAD-7: 7-item Generalized Anxiety Disorder Screener
SWEMWBS: Short Warwich-Edinburgh Mental Wellbeing Scale
EPOCH: Engagement, Perseverance, Optimisim, Connectedness, Happiness
PILS-12: 12-item Purpose in Life Scale
GQ-6: 6-item Gratitude Questionnaire
IPTQ: Implicit Personality Theory Questionnaire
MSPSS: Multidimensional Scale of Perceived Social Support
AIM: Acceptability of Internvention Measure
IAM: Intervention Appropriateness Measure
PCSC: Perceived Control Scale for Children
SCSC: Secondary Control Scale for Children
FCS: Fully Conditional Specification
LMICs: Low- and middle-income countries
WHO: World Health Organization
IRB: International Review Board
