# Supplementary figures and images for: Testing the effects of the Shamiri Intervention and its components on anxiety, depression, wellbeing, and academic functioning in Kenyan adolescents: study protocol for a five-arm randomized controlled trial
Source: Trials. 2021 Nov 22;22:829. doi: 10.1186/s13063-021-05736-1 (PMC8607059; doi:10.1186/s13063-021-05736-1)

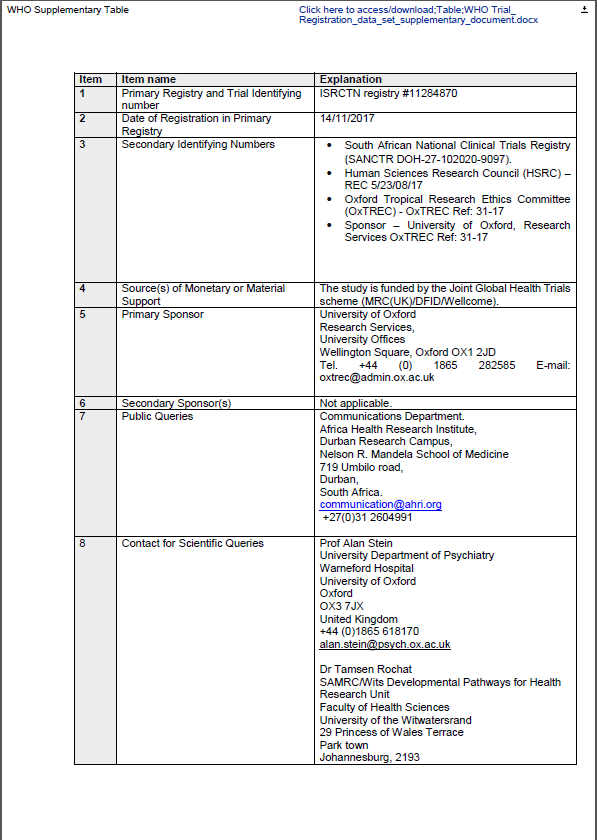

Supplement: Supplementary file 1 — Additional file 1. . [file 13063_2021_5736_MOESM1_ESM.zip › Add1.PNG]

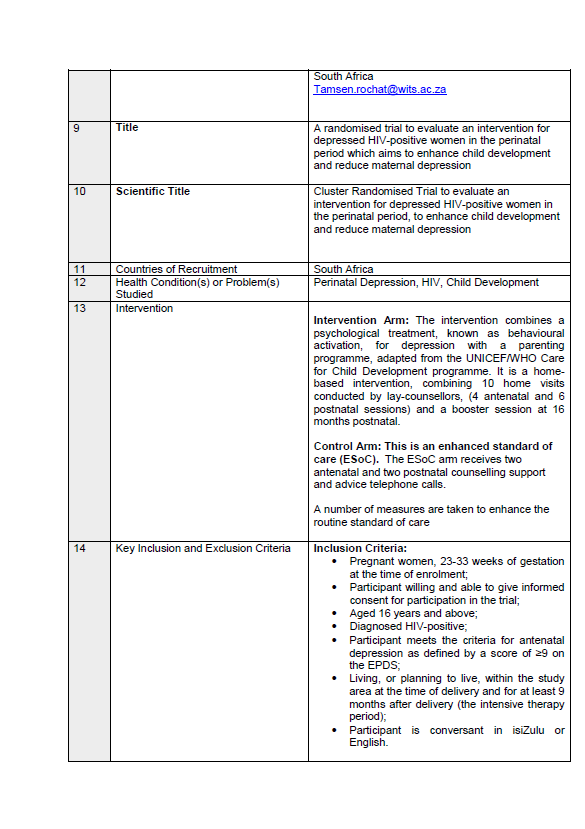

Supplement: Supplementary file 1 — Additional file 1. . [file 13063_2021_5736_MOESM1_ESM.zip › Add2.PNG]

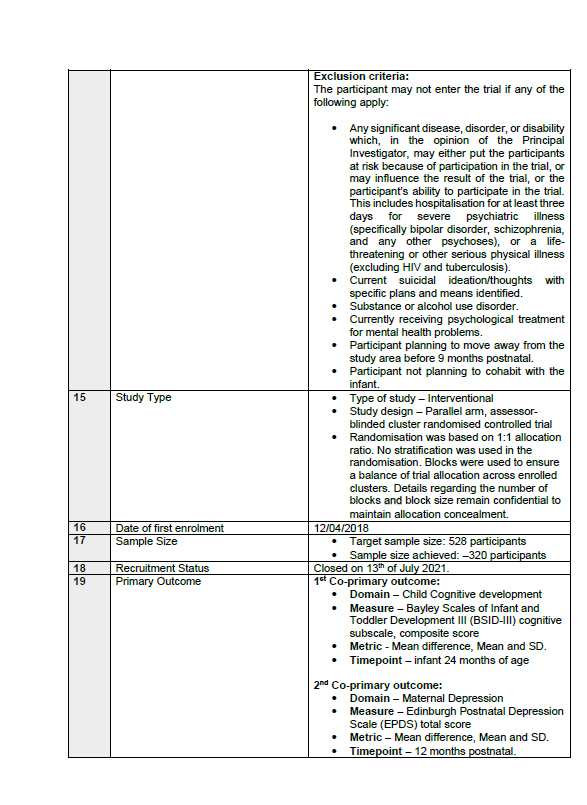

Supplement: Supplementary file 1 — Additional file 1. . [file 13063_2021_5736_MOESM1_ESM.zip › Add3.PNG]

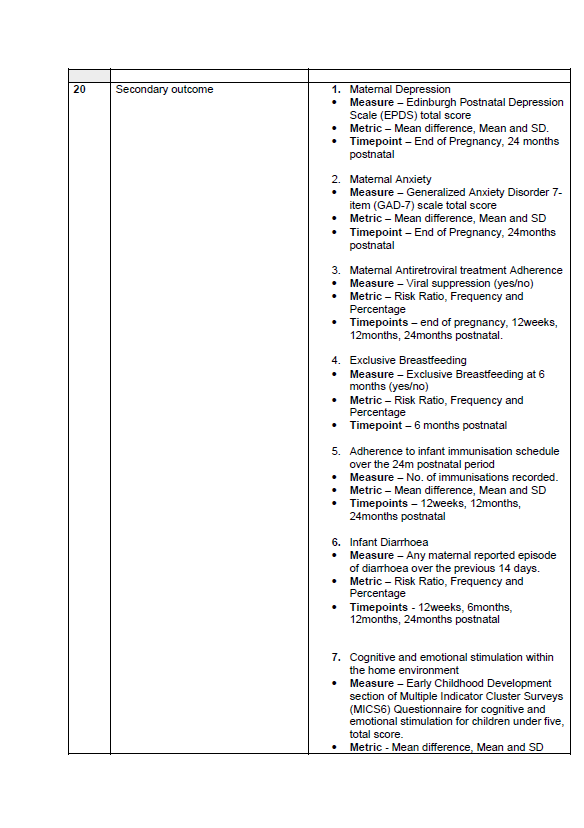

Supplement: Supplementary file 1 — Additional file 1. . [file 13063_2021_5736_MOESM1_ESM.zip › Add4.PNG]

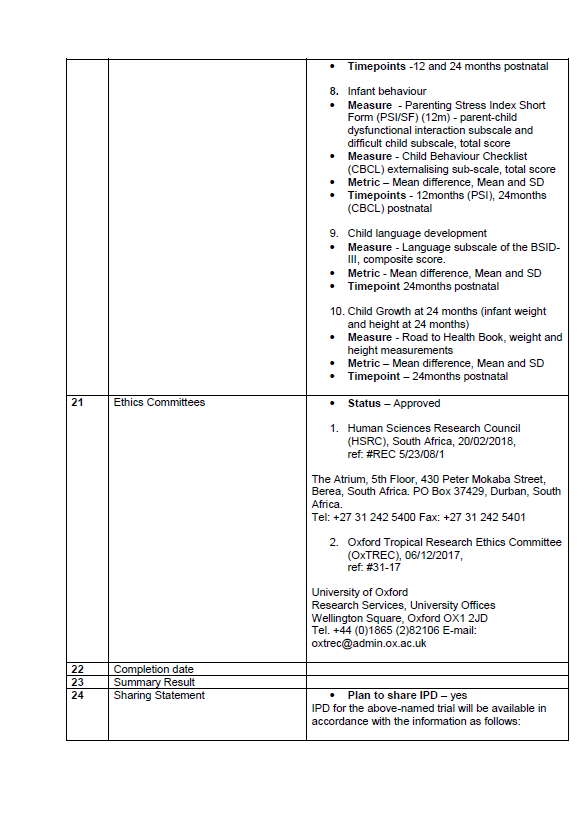

Supplement: Supplementary file 1 — Additional file 1. . [file 13063_2021_5736_MOESM1_ESM.zip › Add5.PNG]

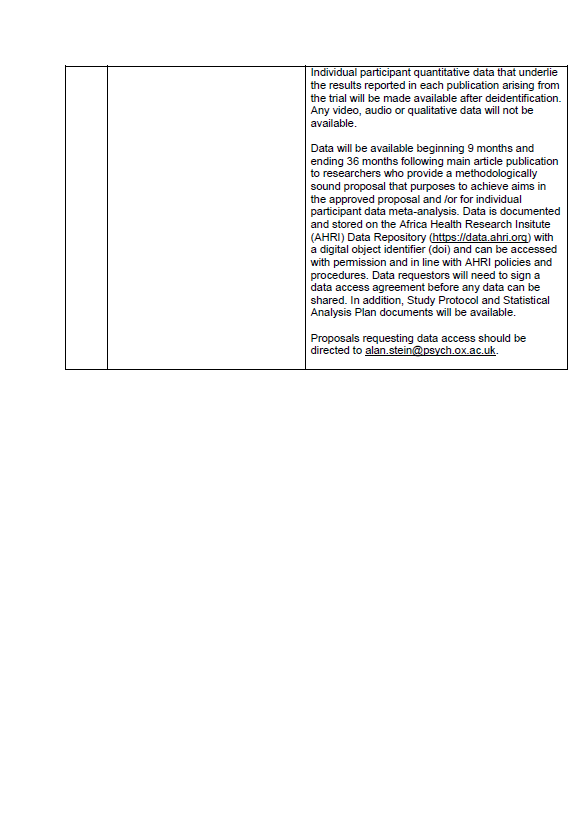

Supplement: Supplementary file 1 — Additional file 1. . [file 13063_2021_5736_MOESM1_ESM.zip › Add6.PNG]
