# Supplementary material for: Testing the effects of the Shamiri Intervention and its components on anxiety, depression, wellbeing, and academic functioning in Kenyan adolescents: study protocol for a five-arm randomized controlled trial
Source: Trials. 2021 Nov 22;22:829. doi: 10.1186/s13063-021-05736-1 (PMC8607059; doi:10.1186/s13063-021-05736-1)
Supplement: Supplementary file 2 — Additional file 2. . [file 13063_2021_5736_MOESM2_ESM.docx]

**INFORMED ASSENT FOR CHILDREN**

**Project Title:** Shamiri: Improving Character Strengths, Wellness, Social Functioning and Academic Achievement in Kenyan High School Youth.

## Protocol Number:

**Research Investigators:**

Dr. Christine M. Wasanga, Project Co-Director, Kenyatta University Dr. John R. Weisz, Project Co-Director, Havard University

Tom L. Osborn, Project Operations Sub-Director, affiliated to Harvard University and Shamiri Institute

Ms. Katherine E. Venturo - Cornerly, Project Scientific Co-Sub-Director, Harvard University, and Shamiri Institute

Mr. Akash Wasil, Project Scientific Co-Sub-Director, University of Pennsylvania.

The investigators named above are doing a research project.

## These are the things we want you to know about research project:

We are asking you to be in a research study. Research is a way to test new ideas. Research helps us learn new things.

Whether or not to be in this research is your choice. You can say Yes or No. Whatever you decide is OK.

## What is the study about?

The purpose of the study is to improve the wellbeing, academic success, social support, and mental health of high school students in Nairobi, and to contribute to research to better a) understand the psychosocial needs of and b) serve at-risk adolescents in Kenya. The significance of this study is that it will provide evidence that brief, positive-psychology focused interventions that are delivered by lay-providers in schools may help improve wellbeing and academic performance of adolescents living in sub-Saharan Africa.

## Why am I being asked to be in this research study?

You are being asked to be in the study because you possess the characteristics that were required for the study participants such as high school student and as we cannot visit all the high schools in Kenya. Therefore, you are representing many other students who we will not be able to reach.

## What will happen during this study?

If you agree to be in this study, you will be divided into groups. Some groups of students will be taught study skills, while the other groups will be taught cognitive and behavioral skills and information about psychology. You will be possibly randomized into one of these groups and therefore, you will not choose which group to belong to.

Groups will meet for at least one hour for four weeks. You will be asked to fill out questionnaires for up to six weeks after the discussion groups are over. These sessions will be audio-recorded.

We may contact you in the future to complete further questionnaires. After the study is completed, identified data will be destroyed.

## Will the study hurt/risks?

There are no risks or you will not be hurt by participating in the study. However, discussions in the groups may sometimes make you feel uncomfortable. Additionally, some questions on the questionnaires that you are asked to fill out may make you uncomfortable or may ask for information which you do not wish to share. If that is the case, you may refuse to answer the question or choose to leave the study.

Another possible risk is the risk of loss of time. You will spend approximately 1.5 hours per week for six week participating in this study. We expect that the benefits to participant wellbeing and academic performance may outweigh this risk. We will also ensure that you will not miss any class time because of the study.

Finally, there is some risk that you may reveal things in the groups that cause others to think of you or treat you differently. This may cause you to worry and become anxious. If this occurs, peer group leaders will reach out to school administrators as appropriate and defer to your school’s standard procedures. Additionally, group leaders may be able to consult with a professional psychologist to determine the way forward. Furthermore, you should feel free to reach out to your group leader if you think it is necessary. Finally, participants are free to drop out of the study if they are feeling uncomfortable.

## What else should I know about the study?

If you feel sick or afraid that something is wrong with you, speak to your group leader who will know what to do. You do not have to answer any questions that are asked of you.

## What are the good things /benefits that might happen?

During each session as an incentive to attend, a student present will be selected at random through a lottery to receive a small prize (i.e., water bottle, Tshirt or KES 300).

While we cannot guarantee benefits to participants, you may benefit from

participating in our study in a variety of different ways. First, you may notice improvements in your wellbeing and in your academic functioning and grades.

You may learn and reinforce important skills for the future. In addition, they may also feel that they have closer social ties to their classmates because of the groups. More broadly, if successful, the intervention could be used more broadly in Kenya and elsewhere to improve students’ wellness and academic success.

## What if I don’t want to be in this study?

Participation in this research is voluntary, and you can leave the research at any time it will not be held against you. You may ask questions related to the study at any time. You may refuse to respond to any questions and you may stop an interview at any time.

## Who should I ask if I have any questions?

If you have questions about the study call (in most schools, students are not allowed to have phones in schools) Dr. Christine M. Wasanga, 0721355108 or Project Operations sub-Director, Tom Osborn at 0756121145.

However, if you have questions about your rights as a study participant: You may contact Kenyatta University Ethical Review Committee Secretariat on [chairman.kuerc@ku.ac.ke,](mailto:chairman.kuerc@ku.ac.ke) or, [secretary.kuerc@ku.ac.ke](mailto:secretary.kuerc@ku.ac.ke)

## Do I have to be in the study?

No, you do not have to be in the study. Even if you say yes now, you can change your mind later. It is up to you. No one will be mad at you if you don’t want to do this.

## Signatures

Before deciding if you want to be in the study, ask any questions you have. You can also ask questions during the time you are in the study.

If you sign your name or put a mark below, it means that you agree to take part in this research study.

Your Name (Printed) Age

Your Signature Date

Signature of Person Obtaining Consent Date

Signature of Witness Date
