# Supplementary material for: Testing the effects of the Shamiri Intervention and its components on anxiety, depression, wellbeing, and academic functioning in Kenyan adolescents: study protocol for a five-arm randomized controlled trial
Source: Trials. 2021 Nov 22;22:829. doi: 10.1186/s13063-021-05736-1 (PMC8607059; doi:10.1186/s13063-021-05736-1)
Supplement: Supplementary file 3 — Additional file 3. . [file 13063_2021_5736_MOESM3_ESM.docx]

**Shamiri Wellness Program Protocol for Group Leaders (Lay – Providers)**

**Follow the protocol:** Don’t add anything or omit anything (unless you are running short on time, in which case you can cut discussions short by calling on fewer students.)

**Watch the time:** Look at the time allotted for each section, and check your watch, clock, or phone to make sure you don’t run overtime.

**Handling incorrect responses:** Everyone is unique, and we will respect those differences. However, if someone says something clearly incorrect you may: ask others in the group what they think about it, point out the issues with it in a lighthearted way, gently correct the student’s misunderstanding yourself, or offer to speak with the student more after the session if they are still confused or don’t agree with you.

**Handling risk:** Refer to your risk protocol for details. If you are worried that a participant may harm her/himself or others, **speak to your supervisor** as soon as you can (usually right after the session).

**Combined Shamiri Wellness Program Protocol**

**Session 1: Growth Mindset**

- **Required sheets:**
  - 1. Neuroplasticity - the Simple Science of Growth.
  - 2. Growth Testimonials
  - 3. A Time You Grew
  - 4. Growth Take-Home Activity Week 1
- **Session overview:**
  - Part 1: Questionnaires (*5-7 min*)
  - Part 2: Icebreaker *(6-8 min)*
  - Part 3: Introduction *(3 min)*
  - Part 4: Introduce rules and expectations for the group *(3-4 min)*
  - Part 5: Growth Mindset Article & Video *(8-10 min)*
  - Part 6: Growth Mindset Discussion *(5 min)*
  - Part 7: Growth Testimonials *(10-12 min)*
  - Part 8: Assignment: “Write your own growth story” *(6 min)*
  - Part 9: Discussion about growth stories *(5-6 min)*
  - Part 10: Explain the Growth Take-Home Activity *(2 min)*

**Part 1: Questionnaires** *(5-7 min)*

- Pass out the questionnaires
- Hand out pens and notebooks to each student
- Tell students to take about 5 minutes to do the questionnaires
- Remind students before they start the questionnaires:
  - The questionnaires will ask about how they’ve been doing (socially academically, and in terms of wellness)
  - Responses will be kept private (no one except the Shamiri team will see them)
  - No one at the school will see their responses (the administration and teachers will not have access to them)
  - There are no right or wrong answers (you will not be graded)
  - You should answer as honestly as possible
- Answer any questions that students have
- Collect the questionnaires

**Part 2: Icebreaker** *(6-8 min)*

- Introduce yourself and ask everyone’s name (keep this short!)
  - Icebreaker (pick ONE icebreaker activity you think your group will like)
    - Examples:
      - Two truths and a lie (everyone says two true things and one lie about themselves; other group members guess which one is the lie)
      - Guess who wrote it (each person should write down one hobby they have and why they like it; read out the hobbies one by one and have other group members guess who wrote down each hobby)
      - Rosebud/thorn (have everyone go around and say a highlight from their week, a low point of their week, and something they are looking forward to)

**Part 3: Introduction** *(3 min)*

- This program is important because it is designed to:
  - Help students achieve their goals, feel happier, do better in school, and improve their lives
  - Help them learn how to handle problems and improve their overall wellbeing
- The program is designed to improve wellness and academic performance
  - Uses research from **Harvard University** and **Stanford University**
  - This research has **helped students right here in Kenya, and in America and Europe**
  - **Results from the last several years showed that activities like the ones we are going to do helped students from Kenya to feel happier and get better grades**
- Students who participated in this study last year said that it helped them to:
  - Build better social relationships, do better in school, feel happier, have more hope and more skills for the future, learn to better handle challenges, and figure out what is most important to them
- Throughout this program, we will be talking about ways in which each of you can improve your life.
  - So, as we learn over the next few weeks, try to think about how what you learn can apply to you.
  - For example, maybe it can help you improve your family relationships or friendships, achieve your goals, or feel better.

**Part 4: Introduce rules and expectations for the group** *(3-4 min)*

- Ask: **How do you want people to act in the groups? What rules do you want to set?** (Allow 3-4 students to speak, then say other rules; make sure to include the rules below; you may also have other rules that students come up with)
  - **Be respectful of others**
  - Don’t talk over anyone
  - Participate as much as you would like
    - Be open and honest
    - Anything you want to say or not say is okay
  - Carry your pen and notebook to the sessions
  - **We want to hear from all of you – please participate if you feel comfortable**
  - **Confidentiality: Do NOT** **share anyone’s personal information**. *THIS IS IMPORTANT!!!!*
    - Explain how you wouldn’t like it if your secrets were shared, so they shouldn’t share anyone else’s information
- Also, it is important that you not tell others at the school about what you learn, because we are doing a scientific study of this program, and if you tell others what you learned, we might not be able to tell how well the program works. You can talk with people in your school about it in a year when the study is over!
- **We won’t share anything that you say in the groups with anyone else; it will all stay private from everyone else at school. The only situation in which we would have to tell someone else is if you tell us you’re thinking of seriously harming yourself or someone else, in which case we will have to tell someone from the Shamiri team, or possibly from the school. In all other cases, everything will stay private!**
- If you break the rules, we will:
  - Talk with you one-on-one
  - Not allow you to enter the t-shirt raffle
  - If you continue to break the rules, we will remove you from the group and talk with your principal
- Pause and ask if anyone has questions
- Then ask them: Can you commit to following the rules?
  - They should all say “yes” to this.

***~40 min left***

**Part 5: Growth Mindset Article & Video** *(8-10 min)*

- Today’s lesson will be on growth, or personal improvement
  - **Offer a short definition of growth**
    - Example: “Growth is about becoming better at things over time. Sometimes, people assume that their abilities, feelings, actions, and thoughts are fixed, but this is incorrect. In fact, people can change or grow anytime and in many areas of their lives.”
    - Give a few examples of areas in which people can grow (for example: relationships with friends, math, happiness)
  - **Ask students to look at the article on growth and neuroplasticity on page 2 of their booklets: “1. Neuroplasticity - the Simple Science of Growth.”**
    - Ask for a volunteer to read each paragraph
  - **Show the two-minute video on growth and neuroplasticity.** *(2-3 min)*
    - Briefly summarize the article and video in your own words. *(1-2 min).* In your explanation, mention that:
      - Your brain can change
      - You can grow in **many different areas of life (not just intelligence!)**
      - Give **a few examples** of areas in which people can grow (for example kindness, happiness, athletics, patience)

**Part 6: Growth Mindset Discussion** *(5 min)*

- **Lead a discussion about the article and the video**
  - Sample questions:
    - What is the main idea of the article and video?
    - How would you describe growth in your own words?
    - How would you describe neuroplasticity in your own words?
    - How can you apply these ideas to your life?
    - Do you have any questions about growth?
    - Did you learn anything new about growth after the article and video?
- During this discussion, try to:
  - **Validate** and **restate** the parts of students’ answers that accurately describe growth and neuroplasticity
  - **Emphasize** that growth can happen in many ways (for example: intelligence, personality, happiness, friendships, etc.)
  - **Emphasize** that growth requires hard work and time – not magic!
    - Try to tie together the importance of growth, hard work, and success
  - **Emphasize** that failures are part of the process of growing, and you can learn from them
  - **Give examples** if students are having trouble defining growth or neuroplasticity

***~25 min left***

**Part 7: Growth Testimonials** *(10-12 min)*

- You’ll now share some examples of how people have faced failures and challenges and grown as a result.
  - In each of these stories, someone:
    - Faced a challenge or setback
    - Used effort and strategies to overcome the challenge
    - Learned or improved or grew as a result of the challenge
- **Ask volunteers to read the stories of Eunice Mwabe and Kago Kagichiri on page 3 of the booklet (“2. Growth Testimonials”)**
- **Lastly, quickly provide your own growth story (as a group leader)**

**Part 8: Assignment: “Write your own growth story”** *(6 min)*

- Tell the students that they will each write their own growth story
  - **Pass out sheet: “3. A Time You Grew”**
  - Emphasize that students should include three things in their story:
    - The challenge they faced
    - How they used effort or strategies to deal with the challenge, and any setbacks they faced along the way
    - How they learned or improved or grew as a result of the challenge
  - Give them 5 min to finish the sheet

***~8 min left***

**Part 9: Discussion about growth stories** *(5-6 min)*

- **Lead a discussion about the growth stories.**
  - Sample questions:
    - Would some of you please share what you wrote?
    - What strategies did you use to overcome your challenge?
    - Did you face any failures along the way in your growth story?
    - How did it feel completing this activity?
  - During this discussion about personal growth stories, try to:
    - **Validate** and **restate** the three parts of participants’ stories.
      - Example: “Thank you so much for sharing. It seems like you were having trouble in physics (Part 1: Challenge), so you decided to talk with the teacher more and study more (Part 2: Effort/strategies), and now you love physics! (Part 3: Growth).
    - **Emphasize** the parts of the story that involve **effort** and **specific** **strategies**
      - Example: It sounds like the strategies you used were very helpful! You did more practice problems and you spoke with your teacher.
    - **Highlight** that growth does not happen because of “magic”
      - It requires effort, hard work, and strategies, and sometimes it’s not perfect.
    - **Highlight that often, you face failures along the way and you can learn from these failures**
      - Example: first, you tried doing more practice problems. But you didn’t improve immediately because there were some you couldn’t figure out on your own, so you reached out to a friend who helped you with the problems you didn’t understand.

**Part 10: Explain the Growth Take-Home Activity** *(2 min)*

- **Pass out the take-home sheet: “4. Growth Take-Home Activity Week 1”**
- **Ask students to read the instructions aloud**
  - Identify a challenge you face during the next week
  - Write about how effort, strategies, and knowledge about growth can help you handle the challenge
  - Write about how you could grow as a result of the challenge
- Remind students that we will be checking to make sure they did the homework. You can only enter the raffle if you do the homework.
- Ask if anyone has any questions
- Tell them you look forward to seeing them next week

**Session 2: Growth Mindset Part 2**

- **Required Sheets:**
  - 5. What Helps You?
  - 6. Effective Strategy List
  - 7. STEPS for Solving Life’s Problems
  - 8. Letter to a Friend
- **Session Overview:**
  - Part 1: Lead a discussion about the Growth Take Home Activity *(6-8 min)*
  - Part 2:
    - A: Discussion About Strategies Part 1 *(3-4 min)*
    - B: Discussion About Strategies Part 2 *(8 min)*
  - Part 3: Teaching Problem Solving Skills *(10 min)*
  - Part 4: Letter to a Friend *(10 min)*
  - Part 5: Discussion of Letters/Conclusion *(5 min)*
  - Part 6: Problem Solving HW (“6. STEPS for Solving Life’s Problems”) *(1-2 min)*
  - Part 7: Midpoint Questionnaires *(15 min)*

**Part 1: Lead a discussion about the Growth Take Home Activity** *(6-8 min)*

- Sample questions:
  - Does anyone want to share what challenge they faced, and how they handled it?
  - What strategies or techniques did you use to handle the challenge?
  - What was rewarding about the activity?
  - What was challenging about the activity?
- During this discussion about the HW assignment, try to:
  - **Validate** and **restate** the three parts of participants’ homework
    - Example:
      - “Thank you so much for sharing. It seems like you were having trouble in physics (Part 1: Challenge), so you decided to talk with the teacher more and study more (Part 2: Effort/strategies), and now you think you will see improvement in physics! (Part 3: Growth).
  - **Emphasize** the parts of the story that involve **effort** and **specific** **strategies**
    - Example:
      - It sounds like the strategies you used were very helpful! You did more practice problems and you spoke with your teacher.
  - **Highlight** that growth does not happen because of “magic”.
    - It requires effort, hard work, and strategies, and sometimes it’s not perfect.
  - **Highlight that often, you face failures along the way and you can learn from these failures**
    - Example:
      - First, you tried doing more practice problems. But you didn’t improve immediately because there were some you couldn’t figure out on your own, so you reached out to a friend who helped you with the problems you didn’t understand.

**Part 2A: Discussion About Strategies Part 1** *(3-4 min)*

- Tell the students that you’re going to ask them to think about what strategies they use to grow and overcome challenges.
- **Pass out the sheet: “5. What helps you?”**
- Ask the students to read the instructions aloud.
- Then, ask the students to write down a few strategies on their own on the sheet *(2-3 min)*
  - These strategies can be things that they do when they feel upset, when they have a conflict with another person, or things that they do to improve their academics *(2-3 min)*

**Part 2B: Discussion About Strategies Part 2** *(8 min)*

- Next, lead the discussion by asking people to share what they wrote down.
- Sample questions for the discussion:
  - When you feel upset, what types of things do you do to feel better?
  - If you’re worried about something, what do you to relax?
  - If you’re feeling sad, what might you do to cheer yourself up?
  - When you’re worried about an assignment or an exam, what strategies do you use?
  - If you have a conflict with someone else, what might you do to improve the situation?
- During this discussion, try to:
  - **Ask follow-up questions** to get students to be as **specific** as possible.
    - Example:
      - So when you’re upset, it helps when you talk with your friends. What do you usually say to your friends? Do you talk about the thing that’s bothering you, or something else? How do you decide which friends to talk to?
  - **Validate** and **relate to** some of the strategies. If possible, make their strategies more specific or occasionally suggest extra strategies.
  - **Refer** to the sheet **(“5. What Helps You?”)** if you want to add some examples to the list.
    - Example:
      - “Yeah, reaching out to friends can be really helpful. Sometimes, I even ask them to go on a walk with me. That way, I get to talk with them and I also move around a bit.”
    - Example:
      - “Yeah, writing about the problem can be really nice. One of my friends told me that she thinks about how someone she admires would handle the problem.”
- **Pass out the sheet: “6. Effective Strategies List” for the students to look at and learn some other helpful strategies.**

***~40 min left***

**Part 3: Teaching Problem Solving Skills** *(10 min)*

- **Pass out sheet: “7. STEPS for Solving Life’s Problems”**
  - Now we are going to talk more about how you can grow.
  - You may be wondering how you should work to solve life’s problems. We will discuss steps you can follow for problem-solving now.
- **Ask: Will each of you read one of the steps from the sheet?**
- As they read, briefly explain each of the STEPS:
  - S: Say What the Problem Is:
    - Figure out exactly what the problem is you want to solve
  - T: Think of Solutions:
    - Make a list of many possible solutions even if you don’t think you’ll use all of them
  - E: Examine Each One:
    - Positives and Negatives
      - Think about the things you like and don’t like about each solution you thought of
  - P: Pick One:
    - Pick the best solution for you
  - S: See if it Works:
    - Consider what might go wrong when trying that solution and how you could handle these issues
    - This may sometimes mean selecting another solution
  - Finally:
    - Try the solution:
      - Do the thing you chose to do, and evaluate how it went, then go through the steps again if you need to
- *(Optional if there is time)*: **Group leaders should share a simple problem they are facing (can be made up)**
  - Example:
    - You’ve been arguing with your sister over doing enough chores.
    - Ask the students to walk through the steps to solve the problem with you *(3-4 min)*
- Tell them you’ll all fill out the sheet later at home to practice more problem solving

**Part 4: Letter to a Friend** *(10 min)*

- So far, we’ve learned about neuroplasticity and how our brains can grow, we’ve brainstormed some strategies that can help us, and we have talked about steps for solving problems. Now, we’re going to try to use what we learned in order to help others.
  - **Give students the sheet: “8. Letter to a Friend”**
- **Tell students to read the instructions aloud.**
- Let the students know that the letter will remain confidential. No one will see it except a few people in the Shamiri team.
- Ask if they have any questions about the prompt.

***~20 min left***

**Part 5: Discussion of Letters/Conclusion** *(5 min)*

- Lead a wrap-up discussion about growth. This discussion should start with the letter to a friend activity, but perhaps move onto more general questions if it seems best.
  - **Sample questions:**
    - Would someone like to share what they wrote in their letter to a friend? (Allow students to share until it seems they’re ready to move onto the questions below.)
  - **Other questions:**
    - How can you use the ideas we’ve learned about in your lives?
    - Are there any strategies you want to use this week?
    - How can the stuff we talked about help you in your academics?
    - How can the stuff we learned about help you in your relationships?
    - How can the stuff we learned about help you overcome challenges?
- **In this discussion, emphasize** how **you can grow in many different aspects of life**, including happiness, friendships and family relationships, and intelligence.
- **Emphasize the importance of effort and strategies.**
- **Emphasize the importance of failure for growth.**

**Part 6: Problem Solving HW (“6. STEPS for Solving Life’s Problems”)** *(1-2 min)*

- Ask students to think of a problem of their own that they could use problem solving to help solve; it should be something that is bothering them or has been on their mind
- Remind everyone to try and pick a problem that they have some control over and that isn’t too huge. For example, it is too hard to create world peace in a week!
- **Fill out the sheet for their own problem for HW**
- Try out the solution they decide on this week and see how it goes

**Part 7: Midpoint Questionnaires** *(15 min)*

- Pass out the questionnaires
- Tell students to take about 10 minutes to fill them out
- **Remind students before they fill out the measures:**
  - Their responses will be kept private (no one except the Shamiri team will see them)
  - No one at the school will see their responses – the administration and teachers will not have access to them
  - There are no right or wrong answers; they will not be graded
  - Please answer honestly
- Answer any questions that students have

**Session 3: Gratitude**

- **Required sheets:**
  - 9. Gratitude Letter
  - 10. What am I Grateful for about Myself?
  - 11. Three Good Things
- **Session Overview:**
  - Part 1: Discussion of Problem Solving HW *(10 min)*
  - Part 2: Explain What Gratitude Is *(4 min)*
  - Part 3: Discussion about Gratitude *(15 min)*
  - Part 4: Gratitude Letter *(15 min)*
  - Part 5: What am I Grateful for about Myself? *(10 min)*
  - Part 6: Three Good Things HW *(3-4 min)*

**Part 1: Discussion of Problem Solving HW** *(10 min)*

- What problem did you choose to try and solve this past week?
- How did you go about identifying the problem?
- What solution did you pick, and why did you pick the one you did?
- How did it go when you tried to act on your solution?
- Did you encounter any obstacles? If so, how could you pick a different solution or get around them in the future?

**Part 2: Explain What Gratitude Is** *(4 min)*

- Today, we’re going to talk about gratitude.
- **Gratitude is noticing good things in your life and appreciating them.**
- This week, we’re going to identify good things in our lives that we are thankful for and talk about how to notice and express our gratitude.
- You can be grateful for anyone and anything.
- Give a personal example of something you’re grateful for.
  - Example: I am thankful for my friends and for chocolate.
- It is important to notice, say, and think of things we’re grateful for.
  - Research shows that practicing gratitude makes you happier and improves sleep and relationships.
- It’s not always easy to feel grateful. However, you can change the way your brain processes gratitude through practice, making it easier and easier to experience the benefits of gratitude.
- Ask if anyone has any questions.

***~45 min left***

**Part 3: Discussion about Gratitude** *(15 min)*

- Can any of you describe gratitude in your own words?
- **Validate** aspects of their responses that you think are helpful and correct.
- What/whom in your life are you grateful for? Why are you grateful for these things/people? **Have everyone answer. Go around in a circle.**
- What has happened in the past few days that has made you feel happy? What good things have happened to you recently? **Have everyone answer. Go around in a circle.**

***~30 min left***

**Part 4: Gratitude Letter** *(15 min)*

- Make sure everyone has the sheet: “**9. Gratitude Letter**”
- Explain to the students that they should think of one person to write a letter to who:
  - Did something for them or who helped them in some way
  - They haven’t properly thanked
- In that letter, you should describe what the person did for you, why you’re grateful, and how they changed your life
- **Explain:** They can give the letter to that person later, or they can keep the letter private, or give it to the group leader. We recommend they give it to the person they wrote it about, but they don’t have to.
- **Do not discuss this activity after the students finish.**

***~15 min left***

**Part 5: What am I Grateful for about Myself?** *(10 min)*

- **Pass out activity sheet: “10. What am I Grateful for about Myself?”**
- In this activity, you will get a chance to think about things about yourself that you like or are grateful for.
- We will ask you to think of one thing about yourself that you’re thankful for.
- You’ll then get a chance to reflect on why you are grateful for this thing about yourself.
- **Ask the students to go around and read the activity instructions.**
- **Do not discuss this activity as a group.**

**Part 6: Three Good Things HW** *(3-4 min)*

- **Make sure all students have the sheet: “11. Three Good Things”**
- At the end of each day, write three good things or people that you are grateful for.
- For each good thing, answer ONE of the questions in the second column. You don’t have to answer the same question for each thing.
- Ask the students to go around and read the activity instructions.
- Ask one or two students to share an example of something they might write down on the sheet.
- Remind students to decide on a time when they will do this activity each day.
- For example, they could do it right before going to sleep each day, or right after brushing their teeth in the morning.
- Ask one or two students for an example of a time they could do the activity each day.
- Thank the students for participating and tell them you’ll see them next week.

**Session 4: Value Affirmations**

- **Required Sheets:**
  - 12. What Matters to Me?
  - 13. What’s Important to Me?
- **Session Overview:**
  - Part 1: Introduction to Values *(5-6 min)*
  - Part 2: Testimonial of Value Affirmations *(2-3 min)*
  - Part 3: Importance of Values Discussion *(8 min)*
  - Part 4: Value Affirmations Writing Prompt *(15 min)*
  - Part 5: Closing Reflection from the Whole 4-Week Program *(5-7 min)*
  - Part 6: Endpoint measures (including feedback) *(20-25 min)*

**Part 1: Introduction to Values** *(5-6 min)*

- **Hand out sheet: “12. What Matters to Me?”**
  - **Describe what values are:**
    - Characteristics (good characteristics!) that are particularly important to a person
    - And help guide a person’s actions and how a person lives
    - Everyone has different values; they are personal
  - **Note that values are similar to virtues,** which you may have learned about before.
  - Give some examples of possible values, using your own values
    - Example: “For example, one of my values is kindness. I want to be kind, especially to my friends. Helpfulness is also one of my values. I want to be helpful, even to strangers. I also value being a strong student and being a helpful group leader. Everyone has different values, different things that are important to them.”
  - Add that it’s okay if you don’t yet know your values. We will be doing some exercises to help you identify them.
  - Add that it is normal for your values to change over time, and you might develop new values in the future.
- **Explain**: The list of values we handed out is not complete; students can add other values if something is missing that is important to them *(2 min)*
- **Activity: Circle the 3-5 values that are most important to you and your life** *(3-5 min)*

**Part 2: Testimonial of Value Affirmations** *(2-3 min)*

- **Group leaders present one story of a role model.** They emphasize three key elements in this story*:*
  - Which key value the person chose and lived by
  - How this value guided their life and decisions
  - How it allowed them to be successful AND live a happy life

**Part 3: Importance of Values Discussion** *(8 min)*

- Ask the students to break into groups of two with the person next to them
- **Discuss in the small groups** any other examples of a role model whose virtues helped them be successful AND achieve happiness in life *(3 min)*
  - **Discuss as a full group:**
    - Would any of you share the role models you discussed with your partner? *(4 min total)*
    - **Reinforce** the key elements of each story
      - For example: Picking a value, using it to help you decide how to live your life, and becoming more successful and happier as a result.

***~45 min left***

**Part 4: Value Affirmations Writing Prompt** *(15 min)*

- **Pass out the sheet: “13. What’s Important to Me?”**
  - Group leaders explain writing task to students: students have 15 min to choose **one** of their three virtues and write about a time when they really demonstrated that virtue.
  - **Ask students to go around and read the instructions aloud**
  - Remind them:
    - Keep in mind what they learned about growth and neuroplasticity as they work toward their goals and values.
    - They can use problem solving to help them achieve their goals.
  - Say: You should not worry about grammar or writing quality, because it is not going to be graded.
  - Take ~12 minutes to do the activity, and do NOT discuss students’ answers.

**Part 5: Closing Reflection from the Whole 4-Week Program** *(5-7 min)*

- Ask everyone to go around and each say one thing they will use in the future or that they learned from the groups AS A WHOLE, not just the last session.

***~25 min left***

**Part 6: Endpoint measures (including feedback)** *(20-25 min)*

- **Pass out the questionnaires**
- Tell students to take about 20 minutes to fill them out
- Remind students before they fill out the document:
  - Their responses will be kept private (no one except the Shamiri team will see them)
  - No one at the school will see their responses – the administration and teachers will not have access to them
  - There are no right or wrong answers; they will not be graded
  - You should answer as honestly as possible
- Answer any questions that students have.
- Explain that this will be the last part of the program.
- Tell your students that you enjoyed working with them and thank them for being good students.
- When the students are finished, collect their questionnaires.
