# Supplementary material for: Testing the effects of the Shamiri Intervention and its components on anxiety, depression, wellbeing, and academic functioning in Kenyan adolescents: study protocol for a five-arm randomized controlled trial
Source: Trials. 2021 Nov 22;22:829. doi: 10.1186/s13063-021-05736-1 (PMC8607059; doi:10.1186/s13063-021-05736-1)
Supplement: Supplementary file 4 — Additional file 4. . [file 13063_2021_5736_MOESM4_ESM.docx]

**Shamiri Study Skills Program Protocol for Group Leaders (Lay – providers)**

**Follow the protocol:** Don’t add anything or omit anything (unless you are running short on time, in which case you can cut discussions short by calling on fewer students.)

**Watch the time:** Look at the time allotted for each section, and check your watch, clock, or phone to make sure you don’t run overtime.

**Handling incorrect responses:** Everyone is unique, and we will respect those differences. However, if someone says something clearly incorrect you may: ask others in the group what they think about it, point out the issues with it in a lighthearted way, gently correct the student’s misunderstanding yourself, or offer to speak with the student more after the session if they are still confused or don’t agree with you.

**Handling risk:** Refer to your risk protocol for details. If you are worried that a participant may harm her/himself or others, speak to your supervisor as soon as you can (usually right after the session).

**Shamiri Academic Skills Program Protocol**

**Session 1: Note-Taking**

- **Required sheets:**
  - 1. Notetaking Worksheet
  - 2. 5 Rs of Notetaking Worksheet
  - 3. Notetaking Pair Share
  - 4. Notetaking Take-Home Activity 1
- **Session overview:**
  - Part 1: Questionnaires *(10 min)*
  - Part 2: Icebreaker *(15 min)*
  - Part 3: Introduction *(10 min)*
  - Part 4: Note-Taking *(8-10 min)*
  - Part 5: Note-taking Pair-Share *(8 min)*
  - Part 6: Note-Taking Discussion *(10-15 min)*
  - Part 7: Explain the Note-Taking Take-Home Activity #1 *(2-3 min)*

**Part 1: Questionnaires** *(10 min)*

- Pass out the questionnaires.
- Hand out pens and notebooks to each student.
- Tell students to take about 5 minutes to do the questionnaires.
- Remind students before they start the questionnaires:
  - The questionnaires will ask about how you’ve been doing (socially academically, and in terms of wellness).
  - Responses will be kept private (no one except the study team will see them).
  - No one at the school will see your responses – the administration and teachers will not have access to them.
  - There are no right or wrong answers; you will not be graded.
  - You should answer as honestly as possible.
- Answer any questions that students have.
- Collect the questionnaires.

**Part 2: Icebreaker** *(15 min)*

- Introduce yourself and ask everyone’s name (keep this short!)
  - Icebreaker (pick one or two ice breaker activities you think your group will like)
    - Examples:
      - Two truths and a lie (everyone says two true things and one lie about themselves; other group members guess which one is the lie)
      - Guess who wrote it (each person should write down one hobby they have and why they like it; Read out the hobbies one by one and have other group members guess who wrote down each hobby)
      - Rosebud/thorn (have everyone go around and say a highlight from their week, a low point of their week, and something they are looking forward to)

***~50 min left***

**Part 3: Introduction** *(10 min)*

- This program is important because it is designed to:
  - Help students achieve their goals, do better in school, and improve their lives.
- Introduce rules and expectations for the group:
  - Ask: **How do you want people to act in the groups? What rules do you want to set?** (Allow 3-4 students to speak, then say other rules; make sure to include the rules below; you may also have other rules)
    - Be respectful of others
    - Don’t talk over anyone
    - Participate as much as you would like
      - Be open and honest
      - Anything you want to say or not say is okay
    - Carry your pen and notebook to the sessions.
    - **We want to hear from all of you – please participate if you feel comfortable**
    - **Confidentiality: Do NOT** **share anyone’s personal information**. *THIS IS IMPORTANT!!!!*
      - Explain how you wouldn’t like it if your secrets were shared, so they shouldn’t share anyone else’s information
      - We won’t share anything you tell us unless you tell us you’re going to hurt someone.
- Also, it is important that you not tell others at the school about what you learn, because we are doing a scientific study of this program, and if you tell others what you learned, we might not be able to tell how well the program works. You can talk with people in your school about it in a year when the study is over!
- If you break the rules, we will:
  - Talk with you one-on-one
  - Not allow you to enter the t-shirt raffle
  - If you continue to break the rules, we will remove you from the group and talk with your principal
- Pause and ask if anyone has questions
- Then, ask them to all: Can you commit to following the rules?
  - They should all say “yes” to this.

***~40 min left***

**Part 4: Note-Taking** *(8-10 min)*

- **Show the Study Skills Video**
- Briefly explain that today’s lesson will be on note-taking
  - Ask: How do you currently take notes?
  - **Say that we are going to talk about five steps for taking effective notes** (the 5 Rs of notetaking)
  - Pass out the “**1. Notetaking Worksheet**”
  - Explain that you can easily divide a typical sheet of paper in the same way as the sheet.
- **Pass out the** “**2. 5 Rs of Notetaking Worksheet**” **(5 Rs are listed below).**
- Have a student read each section:
  - **Record:** during a class/lecture, record in the main column as many meaningful facts and ideas as you can.
  - **Reduce:** as soon as possible and after the lesson, summarize the main and key ideas in the Key Column. Summarizing clarifies meanings and relationships, reinforces continuity, and strengthens memory.
  - **Questions:** Also, write questions you have in the Key Column. Formulate questions based on the notes in the right-hand column. Writing questions helps to clarify meanings, reveal relationships, establish continuity, and strengthen memory. Also, the writing of questions sets up a perfect stage for exam-studying later.
  - **Recite:** Cover the note-taking column with a sheet of paper. Then, looking at the questions or key-words in the question and key column only, say aloud, in your own words, the answers to the questions, facts, or ideas indicated by the cue-words.
  - **Reflect:** Reflect on the material by asking yourself questions, for example: “What’s the significance of these facts? What principles are they based on? How can I apply them? How do they fit in with what I already know? What’s beyond them? Then, write your summary of that page’s notes in the “Summary” section at the bottom of your page.
  - **Review:** Spend at least ten minutes per class every week reviewing all your previous notes. If you do, you’ll retain a great deal for current use, as well as for the exam.

**Part 5: Note-taking Pair-Share** *(8 min)*

- **Pass out the** “**3. Notetaking Pair Share**” **worksheet**
- Divide the students into pairs. Ask the students to discuss the following questions:
  - How would you describe the 5Rs of notetaking in your own words?
  - In what ways do you think the 5Rs could be helpful?
  - How would you describe your current notetaking strategies?

***~18 min left***

**Part 6: Note-Taking Discussion** *(10-15 min)*

- Reconvene as a group.
- Lead a group discussion about the 5Rs of notetaking *(10-15 min)*
- Sample questions:
  - How would you describe the 5Rs of notetaking in your own words?
  - In what ways do you think the 5Rs could be helpful?
  - How would you describe your current notetaking strategies?
  - Can you apply these principles in your school life?
  - What are some challenges that could get in the way of using the 5Rs?
  - Do you have any questions about 5Rs of notetaking?
- During this discussion, try to:
  - **Validate** and **restate** the parts of students’ answers that accurately describe the 5Rs of notetaking
  - **Emphasize** the 5Rs of notetaking
  - **Give examples** if students are having trouble defining the 5Rs of notetaking

**Part 7: Explain the Note-Taking Take-Home Activity #1** *(2-3 min)*

- Pass out the “**4. Notetaking Take-Home Activity 1**” sheet
- Explain that the homework has three parts:
  - When did they use the note-taking strategies?
  - How did they use the 5Rs in class?
  - How did using the 5Rs affect their experience in class?
- Remind students that we will be checking to make sure they did the homework. You can only enter the raffle if you do the homework!
- Ask if anyone has any questions
- Tell the students you look forward to seeing them next week

**Session 2: Note-Taking Continued**

- **Required sheets:**
  - 5. The Importance of Global Warming
  - 6. Notetaking Take-Home Activity 2
- **Session overview:**
  - Part 1: Discussion About Take-Home Assignment *(8-10 min)*
  - Part 2: Notetaking Exercise *(10-12 min)*
  - Part 3: Discussion About Note Taking Exercise *(10 min)*
  - Part 4: Concluding the Note-taking Strategies Session *(5-10 min)*
  - Part 5: Explain the Note-Taking Take-Home Activity #2 *(2-3 min)*
  - Part 6: Midpoint Questionnaires *(15 min)*

**Part 1: Discussion About Take-Home Assignment** *(8-10 min)*

- Lead a discussion about the Take-Home Assignment. Sample questions:
  - How did the activity go for you?
  - Does someone want to share what classes they used the 5Rs in, and how it worked?
  - What note-taking strategies would you want to use in the future? When might you use them?
  - What was rewarding about the activity?
  - What was challenging about the activity?
- During this discussion, try to:
  - Validate and restate the effective strategies in student’s responses
- *Note: If you’re having trouble getting your students to share, consider:*
  - Give an example of from you own notetaking experience in high school.
  - Ask them to break into groups of two for a few minutes to talk before sharing with the bigger group.

***~60 min left***

**Part 2: Notetaking Exercise** *(10-12 min)*

- Pass out the article “**5. The Importance of Global Warming**”
- Ask the students to read the article “The Importance of Global Warming” and use it to practice note-taking on their sheets.
- Read the article aloud
  - You can ask students to volunteer to read it
- Give the students 4 minutes to finish note-taking.

**Part 3: Discussion About Note Taking Exercise** *(10 min)*

- Lead a discussion about the 5Rs of notetaking activity. Sample questions include:
  - Does anyone want to share what they wrote?
  - What strategies (Rs) did you use?
  - How did it feel completing this activity?
- During this discussion, try to:
  - **Validate** and **restate** the parts of the discussions that highlight on of the Rs.

***~30 min left***

**Part 4: Concluding the Note-taking Strategies Session** *(5-10 min)*

- **Lead a wrap-up discussion about note-taking.** The discussion can include the following questions:
  - How can you use these strategies in your life?
  - Are there any specific strategies that you want to use this week?
  - Are there any strategies that you think cannot work for you, and if so, why?

**Part 5: Explain the Note-Taking Take-Home Activity #2** *(2-3 min)*

- Pass out the “**6. Notetaking Take Home Activity 2**” and explain the HW assignment. It is like last week’s but it’s a little different. It has three parts:
  - Identify a specific course that you find challenging.
  - Pick one note-taking strategy that you believe will help you through the course.
  - Write down the note-taking strategy you plan to use and how you will use it to handle the challenge.
- Remind students that we will be checking to make sure they did the homework. You can only enter the raffle if you do the homework!
- Ask if anyone has any questions
- Tell students you look forward to seeing them next week

***~15 min left***

**Part 6: Midpoint Questionnaires** *(15 min)*

- Pass out the questionnaires
- Hand-out pens and notebooks to each student.
- Tell students to take about 10-15 minutes to fill them out
- Remind students before they fill out the measures:
  - Their responses will be kept private (no one except the study team will see them)
  - No one at the school will see their responses – the administration and teachers will not have access to them.
  - There are no right or wrong answers; they will not be graded
  - Please answer honestly
- Answer any questions that students have

# **Session 3: The Study Cycle**

- **Required sheets:**
  - 7. The Study Cycle
  - 8. Study Cycle Pair Share
  - 9. Study Cycle Take Home Activity
- **Session overview:**
  - Part 1: Discussion About Take-Home Assignment *(5-10 min)*
  - Part 2: Introducing the Study Cycle *(2 min)*
  - Part 3: Introduction of the Study Cycle *(10-15 min)*
  - Part 4: Study Cycle Pair-Share *(8-10 min)*
  - Part 5: Study Cycle Discussion *(10 min)*
  - Part 6: Your Study Cycle *(5-10 min)*
  - Part 7: Explain the Study Cycle Take-Home Activity *(5 min)*

# **Part 1: Discussion About Take-Home Assignment** *(5-10 min)*

# Lead a discussion about the Take-Home Assignment. Sample questions:

# How did the activity go for you?

# What note-taking strategies would you want to use in the future? When might you use them?

# What was rewarding about the activity?

- - What was challenging about the activity?

# During this discussion, try to:

# **Validate** the things that students share and use the peer counselling strategies

# **Part 2: Introducing the Study Cycle** *(2 min)*

# Explain what the study cycle is

# The study cycle is a 5-step approach to learning designed to help students learn well and in less time.

# It works the way your brain learns best. It reinforces new content many times and helps you feel more confident. The study cycle can be used to learn in any class!

# Pass out the “**7. The Study Cycle**” handout.

***~50 min left***

# **Part 3: Introduction of the Study Cycle** *(10-15 min)*

# Describe that the sheet you passed out lists the five steps for learning important things from your classes.

# Go through the 5-step Study Cycle, asking a student to read each part off the sheet:

# **Preview:** Quickly look over your text and other study materials before class to develop a general idea/picture of what you'll be learning.

# Skim through the chapter, noting all headings, subheadings, bold words, graphs, pictures and summaries. Once you've looked over and understood the general topics and ideas, it will be much easier to remember and learn the important details.

# **Attend:** Attending or going to class should be an obvious step, but some students don't take it seriously enough. Missing even a few classes can harm your studies and the learning process, especially in subjects where you build skills over time such as math. When attending class, it’s important to be engaged and pay attention. Combined with previewing (step 1), attending class will allow you to get more from classes and take better, more concise notes. If you need to miss a class, ask the teacher or another student to review the material with you!

# **Review:** Sometime during the day, take about 10 minutes to review your class notes. This process of review transfers the information you learned during class from your short-term to your long-term memory. It also reinforces new things you learned and increases confidence.

# **Study:** to reinforce the new material you learned during class, and to make sure you thoroughly understand the subject matter being taught, take about 30-50 minutes to review your notes, read your text book, work on problems, or form a study group. As you study, ask yourself questions about 'how' and 'why' certain things are true, and ask 'what if' questions. Don’t forget, repetition is key!

# **Assess:** Reflect on and evaluate your understanding of the material you learned and studied. Ask yourself, "Is the information I'm studying making sense?", "Am I confident with the new material?", "Do I understand the material well enough that I can teach it to someone else?" Assessing your understanding from time to time is an essential aspect of learning.

**Part 4: Study Cycle Pair-Share** *(8-10 min)*

- Pass out the “**8. Study Cycle Pair Share**” worksheet
- Divide the students into pairs. Ask the students to discuss the following questions:
  - How would you describe each step of the study cycle in your own words?
  - In what ways do you think the steps of the study cycle could be helpful?
  - How would you describe your current study habits?

***~25 mins left***

**Part 5: Study Cycle Discussion** *(10 min)*

- Reconvene as a group.
- Lead a group discussion about the 5 study cycle strategies.
- Sample questions:
  - How would you describe the steps of the study cycle in your own words?
  - In what ways do you think the steps of the study cycle could be helpful?
  - How would you describe your current study habits?
  - Can you apply the study cycle steps in your school life?
  - What are some challenges that could get in the way of using the study cycle steps?
  - Do you have any questions about the study cycle steps?
- During this discussion, try to:
  - **Validate** and **restate** the parts of students’ answers that accurately describe the study cycle steps.
  - **Emphasize** the study cycle steps.
  - **Give examples** if students are having trouble defining the study cycle steps.

# **Part 6: Your Study Cycle** *(5-10 min)*

# Ask the students to break into pairs for 5 minutes

# Tell them to, using the study cycle handout, think about how each of the students in the pair could have used the study cycle last week in their classes.

# What would they keep the same that they did last week?

# What would they do differently?

# Reconvene as a group, and ask the students to share what they talked about in their small groups.

***~5 mins left***

**Part 7: Explain the Study Cycle Take-Home Activity** *(5 min)*

- Explain the “**9. Study Cycle Take Home Activity**” assignment.
  - Identify a specific course that you find challenging.
  - Pick one study cycle strategy that you believe will help you through the course.
  - Write down the study cycle strategy you plan to use and how you will use it to handle the challenge.
- Remind students that we will be checking to make sure they did the homework. You can only enter the raffle if you do the homework!
- Ask if anyone has any questions.
- Tell students you look forward to seeing them next week

# **Session 4: The Study Cycle Continued and Conclusion**

# **Session overview:**

# Part 1: Discussion About Take-Home Study Cycle Assignment *(10-15 min)*

- - Part 2: Conclusion *(10-15 min)*
  - Part 3: Closing Reflection from the Whole 4-week Program *(8-10 min)*

# Part 4: Endpoint Questionnaires *(20-25 min)*

# **Part 1: Discussion About Take-Home Study Cycle Assignment** *(10-15 min)*

# Lead a discussion about the Take-Home Assignment. Sample questions:

# How did the activity go for you?

- - What study cycle strategies would you want to use in the future? When might you use them?
  - What was rewarding about the activity?
  - What was challenging about the activity?

# During this discussion, try to:

# **Validate** the things that students share and use the peer counselling strategies

***~50 min left***

**Part 2: Conclusion** *(10-15 min)*

- Lead a wrap-up discussion about study skills.  This discussion will start with a conversation about what people learned, any questions they have, and about how they might use what they learned in the future.
  - Ask: We have talked a lot about strategies to help you improve academically. Do you have any questions about anything you learned?
- Then, if you have time, lead a broader discussion about how the students will use what they learned.
  - Sample questions:
    - What were your favorite parts of this program?
    - What did you think of the program?
    - What are some strategies from the program that you will continue to use?

# During this discussion, try to:

# Validate the things that students share.

# Highlight strategies and lessons that students can continue to use in their lives

***~35 min left***

**Part 3: Closing Reflection from the Whole 4-week Program** *(8-10 min)*

- Ask everyone to go around and each say one main thing they learned from the groups AS A WHOLE, not just the last session.

# **Part 4: Endpoint Questionnaires** *(20-25 min)*

- Pass out the questionnaires
- Tell students to take about 20 mins to fill them out
- No one will know what they said
  - Their responses will be kept private (no one except the study team will see them)
  - No one at the school will see their responses – the administration and teachers will not have access to them.
  - There are no right or wrong answers; they will not be graded
  - You should answer as honestly as possible.
- Explain that this will be the last part of the program.
- Tell your students that you enjoyed working with them and thank them for being good students.
- When the students are finished, collect their questionnaires.
