# Supplementary material for: Testing the effects of the Shamiri Intervention and its components on anxiety, depression, wellbeing, and academic functioning in Kenyan adolescents: study protocol for a five-arm randomized controlled trial
Source: Trials. 2021 Nov 22;22:829. doi: 10.1186/s13063-021-05736-1 (PMC8607059; doi:10.1186/s13063-021-05736-1)
Supplement: Supplementary file 5 — Additional file 5. . [file 13063_2021_5736_MOESM5_ESM.docx]

***Study Team Overview of Emergency Protocol:***

*Risk Guideline Literature Review:* This Emergency Protocol for participant risk scenarios was developed using the American Academy of Pediatrics guidelines for child and adolescent suicide risk, which encourage explicitly asking about suicidal thoughts, intent, plans, and history, as well as assessing risk and protective factors and triangulating interview findings with symptom measures (Shain, 2016). These guidelines also suggest sensitivity and caution and having a natural conversation about how the youth is doing, not just firing off a series of questions about suicide (Shain, 2016). Additionally, related guidelines from the American Academy of Child and Adolescent Psychiatry suggest balancing participant confidentiality and comfort with the need to inform guardians (i.e., teachers in the case of this present study) of child or adolescent risk. In all cases in which harm may be imminent (i.e., high risk) it is necessary to inform the guardian of youth risk (Sondheimer, 2010; Shain, 2016), however, in cases in which the harm is not intended or imminent, steps are less clear-cut and must involve balancing participant comfort and confidentiality with safety, where safety is, of course, most important (Sondheimer, 2010). In an effort to achieve the right balance, we will inform school staff of students who our assessment identifies as posing medium or high risk of suicide; we will not *routinely* inform schools of low-risk cases, but our expert clinician will consider that step in low-risk cases if it seems appropriate. Of course, in all low-risk cases, we will continue monitoring and evaluation and inform the school if the student rises to a higher risk level.

*Study Procedures:* The study staff (including lay-providers) will not systematically ask participants about risk in any questionnaires or in the group meetings. This is in order to avoid unnecessary stigma and discomfort. However, in the event that a student displays potentially serious mental health concerns (e.g., if a participant mentions thoughts of/desire to harm self or others), peer group leaders will be trained to follow-up with the student privately and promptly (e.g., right after the group session) to further assess the situation—for example, by asking about thoughts of harm, and whatever other concerns the student has mentioned. Afterward, as per the training we will provide, group leaders will promptly speak with their supervisor. See emergency protocol for group leaders for more details.

That supervisor will then assist with triage (by asking about thoughts of harm, and, depending on the response, methods, plans, intent, and history). If appropriate, the supervisor will organize follow-up and referral to appropriate resources. All supervisors will be trained to carefully and systematically triage level of risk through a private conversation with the individual who indicated potentially serious concerns. All such conversations, even those that yield a designation of “no risk,” will be reported promptly to study staff including either Tom Osborn or Katherine Venturo-Conerly, and always Dr. Wasanga. See emergency protocol for supervisors for more details.

The possible levels of risk and actions taken for each level of risk are listed below:

- No risk -- for those with no current thoughts of harm to self or others.
  - In these cases, no further monitoring or follow-up will be necessary.
  - Note that this will be the designation for participants who say they used to feel suicidal and do not have any remaining thoughts, etc. of suicide.
- Low-risk -- for those with only thoughts of harm to self or others, and no history of attempt, and no specific methods, plans, or intent.
  - In these cases, study staff will continue to monitor this student through the intervention period, will consult with Dr. Christine Wasanga, who is a study PI and a trained counseling psychologist, and will offer resources to the student as recommended by Dr. Wasanga.
  - Because the student will be monitored throughout the intervention period, if they do at some point reach medium or high risk, school staff will be notified, and the student will be very strongly encouraged to seek individual professional care.
- Medium risk -- for those with thoughts of harm to self or others + any plans (e.g., place, method, time, final preparations) or history of attempt over six months prior (i.e., more than six months prior).
  - In these cases, study staff will continue to monitor this student through the intervention period, will consult with Dr. Christine Wasanga, who is a study PI and a trained counseling psychologist, and will offer resources to the student as recommended by the study PI. Additionally, in these cases, the study staff will make every attempt to have the student speak one-on-one with Dr. Wasanga, either in person or over the phone. Dr. Wasanga will then determine the most appropriate course of action based on her expert clinical judgment. She may recommend measures including, but not limited to, confiscation of means, consultation with school administrators, referral to psychiatrists or psychologists, and involvement of a trusted teacher, parent, or administrator.
  - If Dr. Wasanga deems a student to be at this risk level, Dr. Wasanga will report the student risk to the school, which is the student’s current guardian.
  - At this risk level, we will also make every effort to encourage the student to have psychotherapy sessions with a local mental health professional.
- High-risk -- for those with thoughts + plans + intent or thoughts + recent (less than six months prior) history of attempt.
  - In these cases, study staff will immediately consult with Dr. Christine Wasanga, who is a study PI and a trained counseling psychologist. Then, we will require that the student speak one-on-one with Dr. Christine Wasanga, either in person or over the phone. Dr. Wasanga will then determine the most appropriate course of action based on her expert clinical judgment. She may recommend measures including but not limited to: confiscation of means that could be used to harm self or others, consultation with school administrators, referral to psychiatrists or psychologists, and involvement of a trusted teacher, parent, or administrator. In cases of imminent threat to health and safety, the study team may call on local authorities and customs as needed to help ensure the health and safety of participants and staff.
  - If Dr. Wasanga deems a student to be at the high-risk level, Dr. Wasanga will report the student risk to the school, which is the student’s current guardian.
  - At this risk level, we will also make every effort to encourage the student to have psychotherapy sessions with a local mental health professional.

Professor John Weisz will also be available on call should need be.

If less urgent pressing problems are uncovered in the risk assessment, we may, after consultation with Dr. Wasanga (a counseling psychologist) and Dr. Weisz (a clinical psychologist), refer these students to the appropriate school administrator (e.g., school chaplain, school counselor, school principal). In these cases, we will follow the standard referral procedures used by these schools. For cases in which the study PI is unsure about the need to consult the administration or is unsure about other potential steps, we will contact the Kenyatta University IRB to inquire about an appropriate path forward.

**References**

Shain, B. (2016). Suicide and Suicide Attempts in Adolescents. Pediatrics (Evanston), 138(1),

E20161420.

Sondheimer, A. (2010). Ethics and Risk Management in Administrative Child and Adolescent

Psychiatry. Child and Adolescent Psychiatric Clinics of North America, 19(1), 115-129.

**Emergency Protocol: Shamiri Supervisors**

This document provides a protocol for assessing student risk when you or the group leaders are worried about a student in the groups.

Remember your peer counseling skills during this conversation and be respectful of the student’s privacy by not saying or doing anything to call unnecessarily attention to the situation. Try to have a natural and sensitive conversation, and ask questions in addition to those on this sheet as appropriate to explore students’ feelings. This is a guide including all the essential elements of a risk assessment, but every conversation goes differently, so you may add some things or do things in a different order – that is fine.

1. The group leader you supervise should, when you are called over to do a risk assessment, briefly tell you about why you’ve been called over, which will help you prepare for the conversation.
   1. For example, the group leader might say, “I think it would be very helpful for you to have a conversation with [STUDENT’S NAME] because she/he told me she’s/he’s having a very hard time right now, and you have special training for helping people who are having a hard time.”
2. Introduce yourself, and express interest in and care for the student.
   1. For example: “My name is ____, and I work as a supervisor for your group leader, ____. It sounds like you might be going through a tough time, and I’m here to help make sure that you are safe and cared for.”
3. Explain that you’re going to ask some questions about how the student is doing, so you can better understand and help.
4. First, ask the student generally what has been bothering them, or what this tough time has been like for them. Listen and ask clarifying questions as appropriate.
5. At some point in your conversation, you MUST assess risk of harm to self or others. Get answers to the set of questions detailed here:

**Risk Assessment:**

1. In the last month, have you wished you were dead or wished you could go to sleep and never wake up?
2. In the last month, have you had any thoughts of killing or hurting yourself or someone else?

If no to both, END RISK ASSESSMENT. Student is designated NO RISK. Finish up talking with the student. Report the conversation to Katherine, Tom, or both within a week (e.g., in a supervision meeting).

If yes to 2, get answers to 3, 4, and 5 below.

If yes to 1 and no to 2, skip to 5 below.

1. Have you thought about how you might do this? For example, what you would use or where you would do it?
2. Do you have any intention of acting on your thoughts?

[If the student mentioned a plan: Do you have any intention of carrying out this plan?]

1. Have you ever done anything, started to do anything, or prepared to do anything to end your life?

[Or, if the student mentioned thoughts of hurting someone else: Have you ever done anything, started to do anything, or prepared to do anything to end that person’s life?]

1. If yes to 5: when did you do that, and what did you do exactly?

**No Risk**

If no to questions 1 and 2, then END RISK ASSESSMENT. Student is designated NO RISK. Finish talking with the student. Report the conversation to Katherine, Tom, or both within a week (e.g., in a supervision meeting).

**Low Risk**

If yes to question 1 or 2, but no to 3, 4, and 5, the student is currently at LOW RISK.

- Thank the student for telling you about this. Tell them you’re glad they told you.
- Tell the student that you’ll ask your supervisor, who is an expert in helping people feel better who are in situations like this, for their advice this week, and come back with a plan for the student next week.
- Tell the student you look forward to talking with them next week, and that you’ll be waiting for them after the group meeting finishes.
- Ask the student to please promise you that they won’t hurt themselves this week, and that they’ll talk with you next week.

After the student leaves, call Dr. Wasanga immediately to explain what happened. If you need to take any immediate action, she will tell you. Otherwise, you’ll discuss this issue in weekly supervision and will have a path forward before the next week.

**Medium Risk**

If yes to 1 or 2 and yes to 3, but no to 4 and 5,

OR if yes to 1 or 2, yes to 5, but no the 3 and 4, and they say they attempted **more than 6 months ago**,

the student is currently at MEDIUM RISK. Do not leave the student until Dr. Wasanga has spoken with the student and communicated a plan to you.

- Thank the student for telling you about this. Tell them you’re glad they told you.
- Tell the student that you care about their safety and wellbeing and you’re worried about them, and you’re going to call your supervisor now (Dr. Wasanga, +254 721 35108), who is part of the Shamiri team and an expert in helping people during tough times like this. Ask the student to talk with the supervisor over the phone now to figure out a good path forward.

Stay present during the conversation between your supervisor and the student. The supervisor will determine a path forward quickly, and tell you what the plan is. You will likely be asked to talk with the student again in future weeks, to get the student to promise you they won’t hurt themselves in the coming week and will meet you next week, and to ask the student to give you any things that they considered using to harm themselves or someone else. You or another member of the study team may also be asked to connect that student with school administration.

**High Risk**

If yes to 1 or 2, yes to 3, and 4 or 5, the student is currently at HIGH RISK. Do not leave the student until Dr. Wasanga has spoken with the student and communicated a plan to you.

- Thank the student for telling you about this. Tell them you’re glad they told you.
- Tell the student that you care about their safety and wellbeing and you’re worried about them, and you’re going to call your supervisor now (Dr. Wasanga, +254 721 35108), who is part of the Shamiri team and an expert in helping people during tough times like this. Ask the student to talk with the supervisor over the phone now to figure out a good path forward.

Stay present during the conversation between your supervisor and the student. The supervisor will determine a path forward quickly, and tell you what the plan is. You will likely be asked to talk with the student again in future weeks, to get the student to promise you they won’t hurt themselves in the coming week and will meet you next week, and to ask the student to give you any things that they considered using to harm themselves or someone else. You or another member of the study team may also be asked to connect that student with school administration.

Note: In cases that you deem Medium or High Risk, Dr. Wasanga will make a final determination about risk level after further conversation and triangulation with questionnaires and risk and protective factors and proceed as appropriate. These guidelines are build based on those of the American Academy of Pediatrics (Shain, 2016) and the American Academy of Child and Adolescent Psychiatry (Sondheimer, 2010) and adapted to be appropriate for the context of Kenyan high schools.

**Emergency Protocol: Group Leaders**

This document provides protocols for two different kinds of situations that may be difficult to handle.

Protocol 1: This protocol should be used if a student privately reaches out to you with a difficult situation or a personal problem. Sometimes, you may be able to handle these conversations easily. But, certain situations might make you very uncomfortable, and you may not feel equipped to deal with them. If a student speaks to you in private about a difficult situation or a personal problem:

1. **Talk to the student**: Have the discussion in private; avoid discussing the situation or problem in front of other students. You should…

- Express that you are willing to help
- Listen, understand and clarify what their problem is
- Validate their feelings; For example, you might communicate that their feelings make sense or that you understand that their problem is troubling to them
- Note: You should not give specific advice; you may not be qualified to do that. Instead, you can express your understanding and empathy, ask them what solutions they have thought of, and support them as they think through their options.
- In a calm and relaxed way, tell them that in the case of a significant threat to the student’s safety or someone else’s safety, you would need to contact your supervisor and the school. (for example, “If you tell me that you plan to hurt yourself or someone else, I will have to tell a supervisor and maybe a school official.”).

If the situation requires further action because you feel it is very serious and/or you feel overwhelmed:

1. **Talk to your Shamiri Supervisor** immediately after speaking with the student. **If the student discussed potential harm to self or others, contact your supervisor before the student leaves**.
2. The Shamiri team will decide if school authorities need to be contacted. If you want to talk with a study team member immediately, Tom can also be reached at **+254 756 121 145**.

**If the situation is of significant concern (threat of harm to student or someone else), call your Shamiri Supervisor over immediately after speaking to the student before the student leaves.** Introduce your supervisor to the student, and briefly explain that you think the supervisor could help the student, and briefly explain the student’s concern.

Protocol 2: If a student expresses something in the group that raises a serious concern (suicidal ideas, ideas about harming someone else), but does not directly say they want to talk to the group leader individually:

1. **Approach the student**: in private (probably right after the group), without exposing your concern to the rest of the group or isolating the student from other students. Try to:

- Mention it´s OK to express concerns and emotions
- Offer the chance to talk to you individually
- In a calm and relaxed way, remind them that the conversation will be private, except that in the case of a significant threat to the student’s safety or someone else’s safety, you would need to contact your supervisor and the school authorities. (for example, “if you tell me that you plan to hurt yourself or someone else, I will have to tell a supervisor and maybe a school official.”).

**If the situation is of significant concern (threat of harm to student or someone else), call your Shamiri Supervisor over immediately after speaking to the student before the student leaves.** Introduce your supervisor to the student, and briefly explain that you think the supervisor could help the student, and briefly explain the student’s concern.

If you are having trouble with a logistical issue (need more pens, do not have the appropriate prompt sheets, are having trouble with commuting) contact your Shamiri Supervisor assigned to assist you in the school visit for that day.

If you are feeling overwhelmed with any other situation (example: an issue with a student), speak with your Shamiri Supervisor when you have a chance.
