# Supplementary material for: Testing the effects of the Shamiri Intervention and its components on anxiety, depression, wellbeing, and academic functioning in Kenyan adolescents: study protocol for a five-arm randomized controlled trial
Source: Trials. 2021 Nov 22;22:829. doi: 10.1186/s13063-021-05736-1 (PMC8607059; doi:10.1186/s13063-021-05736-1)
Supplement: Supplementary file 6 — Additional file 6. . [file 13063_2021_5736_MOESM6_ESM.docx]

**Shamiri Growth-Only Program Protocol for Group Leaders**

**(Lay – Providers)**

**Follow the protocol:** Don’t add anything or omit anything (unless you are running short on time, in which case you can cut discussions short by calling on fewer students.)

**Watch the time:** Look at the time allotted for each section, and check your watch, clock, or phone to make sure you don’t run overtime.

**Handling incorrect responses:** Everyone is unique, and we will respect those differences. However, if someone says something clearly incorrect you may: ask others in the group what they think about it, point out the issues with it in a lighthearted way, gently correct the student’s misunderstanding yourself, or offer to speak with the student more after the session if they are still confused or don’t agree with you.

**Handling risk:** Refer to your risk protocol for details. If you are worried that a participant may harm her/himself or others, speak to your supervisor as soon as you can (usually right after the session).

**Session 1: How do People Grow?**

- **Required sheets:**
  - 1. Neuroplasticity - the Simple Science of Growth.
  - 2. Noticing Growth In Real Life Week 1 HW
- **Session Overview:**
  - Part 1: Questionnaires *(5-10 min)*
  - Part 2: Icebreaker *(8 mins)*
  - Part 3: Introduction *(8 mins)*
  - Part 4: Introduce rules and expectations for the group *(3-4 min)*
  - Part 5: Growth Mindset Article & Video *(10-12 mins)*
  - Part 6: Growth Mindset Discussion *(6-8 mins)*
  - Part 7: Homework: Noticing Growth in Real Life *(3 mins)*

**Part 1: Questionnaires** *(5-10 mins)*

- Pass out the questionnaires
- Hand out pens and notebooks to each student.
- Tell students to take about 5 minutes to do the questionnaires
- Remind students before they start the questionnaires:
  - The questionnaires will ask about how you’ve been doing (socially academically, and in terms of wellness).
  - Responses will be kept private (no one except the study team will see them).
  - No one at the school will see your responses (the administration and teachers will not have access to them)
  - There are no right or wrong answers (you will not be graded)
  - You should answer as honestly as possible.
- Answer any questions that students have
- Collect the questionnaires

**Part 2: Icebreaker** *(8 mins)*

- Introduce yourself and ask everyone’s name (keep this short!)
  - Icebreaker (pick ONE ice breaker activity you think your group will like)
    - Examples:
      - Two truths and a lie (everyone says two true things and one lie about themselves; other group members guess which one is the lie)
      - Guess who wrote it (each person should write down one hobby they have and why they like it; Read out the hobbies one by one and have other group members guess who wrote down each hobby)
      - Rosebud/thorn (have everyone go around and say a highlight from their week, a low point of their week, and something they are looking forward to)

***~35 mins left***

**Part 3: Introduction** *(8 mins)*

- This program is important because it is designed to:
  - - Help students achieve their goals, feel happier, do better in school, and improve their lives.
    - Help them learn how to handle problems and improve their overall wellbeing.
- The program is designed to improve wellness and academic performance
  - Uses research from **Harvard University** and **Stanford University**
  - This research has **helped students right here in Kenya, and in America and Europe**
  - **Results from the last several years showed that activities like the ones we are going to do helped students from Kenya to feel happier and get better grades**
- Students who participated in this study last year said that it helped them to:
  - Build better social relationships, do better in school, feel happier, have more hope and more skills for the future, learn to better handle challenges, and figure out what is most important to them
- Throughout this program, we will be talking about ways in which each of you can improve your life.
  - So, as we learn over the next few weeks, try to think about how what you learn can apply to you.
  - For example, maybe it can help you improve your family relationships or friendships, achieve your goals, or feel better.

**Part 4: Introduce rules and expectations for the group** *(3-4 min)*

- Ask: **How do you want people to act in the groups? What rules do you want to set?** (Allow 3-4 students to speak, then say other rules; make sure to include the rules below; you may also have other rules that students come up with)
  - **Be respectful of others**
  - Don’t talk over anyone
  - Participate as much as you would like
    - Be open and honest
    - Anything you want to say or not say is okay
  - Carry your pen and notebook to the sessions.
  - **We want to hear from all of you – please participate if you feel comfortable**
  - **Confidentiality: Do NOT** **share anyone’s personal information**. *THIS IS IMPORTANT!!!!*
    - Explain how you wouldn’t like it if your secrets were shared, so they shouldn’t share anyone else’s information
- Also, it is important that you not tell others at the school about what you learn, because we are doing a scientific study of this program, and if you tell others what you learned, we might not be able to tell how well the program works.
- **We won’t share anything that you say in the groups with anyone else; It will all stay private from everyone else at school. The only situation in which we would have to tell someone else is if you tell us you’re thinking of seriously harming yourself or someone else, in which case we will have to tell someone from the Shamiri team, or possibly from the school. In all other cases, everything will stay private!**
- If you break the rules, we will:
  - Talk with you one-on-one
  - Not allow you to enter the t-shirt raffle
  - If you continue to break the rules, we will remove you from the group and talk with your principal
- **Pause and ask if anyone has questions**
- **Then, ask them to all: Can you commit to following the rules?**
  - **They should all say “yes” to this.**

***~27 mins left***

**Part 5: Growth Mindset Article & Video** *(10-12 mins)*

- Today’s lesson will be on growth, or personal improvement
  - **Offer a short definition of growth**
    - Example: “Growth is about becoming better at things over time. Sometimes, people assume that their abilities, feelings, actions, and thoughts are fixed, but this is incorrect. In fact, people can change or grow anytime and in many areas of their lives.”
    - Give a few examples of areas in which people can grow (for example: relationships with friends, math, happiness).
  - **Ask students to look at the article on growth and neuroplasticity on page 2 of their booklets “1. Neuroplasticity - the Simple Science of Growth.”**
    - Ask for a volunteer to read each paragraph
  - **Show the two-minute video on growth and neuroplasticity.** *(2-3 minutes)*
    - Briefly summarize the article and video in your own words. (1-2 mins). In your explanation, mention that:
      - Your brain can change
      - You can grow in **many different areas of life (Not just intelligence!)**
      - Give **a few examples** of areas in which people can grow (examples: kindness, happiness, athletics, patience)
    - Spend a little bit more time explaining neuroplasticity. Tell the kids that there are pathways/networks in our brain for everything we do. These networks can grow and, because of that, we can change/grow in each and every aspect of our lives.

***~15 mins left***

**Part 6: Growth Mindset Discussion** *(6-8 mins)*

- **Lead a discussion about the article and the video** *(5 mins)*
  - Sample questions:
    - What is the main idea of the article and video?
    - How would you describe growth in your own words?
    - How would you describe neuroplasticity in your own words?
    - How can you apply these ideas to your life?
    - Do you have any questions about growth?
    - Did you learn anything new about growth after the article and video?
- During this discussion, try to:
  - **Validate** and **restate** the parts of students’ answers that accurately describe growth and neuroplasticity.
  - **Emphasize** that growth can happen in many ways (examples: intelligence, personality, happiness, friendships, etc.)
  - **Emphasize** that growth requires hard work and time – not magic!
    - Try to tie together the importance of growth, hard work, and success
  - **Emphasize** that failures are part of the process of growing, and you can learn from them.
  - **Give examples** if students are having trouble defining growth or neuroplasticity.

**Part 7: Homework: Noticing Growth in Real Life** *(3 mins)*

- Pass out the **2. Noticing Growth in Real Life Week 1 HW**
- During the week, look for one thing that reminds you of what you learned today about growth and neuroplasticity.
  - For example, maybe your friend will describe a way in which they improved at something over time.
  - Or, maybe you’ll notice that you struggled at first with something you are doing in school this week, but that you get better at it with practice.
  - Remember, you can notice growth in all sorts of things, including social relationships, emotions, academics, athletics, and more!
- Write down the thing you notice that shows that people can grow (just a sentence or two is fine).
- Ask if there are any questions about the HW assignment.
- Tell the students you look forward to seeing them at the same time, in the same place next week.

**Session 2: Growth Stories**

- **Required Sheets:**
  - 3. Growth Testimonials
  - 4. A Time You Grew
  - 5. Letter to a Friend
  - 6. Growth Take Home Activity Week 2
- **Session Overview:**
  - Part 1: Lead a discussion about the Noticing Growth in Real Life Activity *(5 mins)*
  - Part 2: Growth Testimonials *(10-12 mins)*
  - Part 3: Assignment “Write your own growth story” *(6-7 mins)*
  - Part 4: Discussion about growth stories *(5-6 mins)*
  - Part 5: Letter to a Friend *(10 mins)*
  - Part 6: Explain the Growth Take-Home Activity *(2 mins)*
  - Part 7: Midpoint Questionnaires *(12 minutes)*

**Part 1: Lead a discussion about the Noticing Growth in Real Life Activity** *(5 mins)*

- Sample questions:
  - - Does someone want to share what they noticed this week that reminded them that people can grow, and that their brains can change?
    - How did the things that you noticed relate to what we talked about last week?
    - Do you have any questions about what we talked about last week?
- During this discussion about the HW assignment, try to:
  - **Validate** and **restate** the examples of growth in real life
    - Example:
      - “Thank you so much for sharing. It seems like you were feeling really sleepy, so you decided to start your studying earlier and ask for help when you didn’t get it, and now you think you are getting a little more sleep each night and feeling better.”
  - **Emphasize** the parts of people’s stories that involve **effort, overcoming setbacks,** and **strategies**
    - Example:
      - It sounds like you didn’t know how to apologize to your friend at first, but after you took some time to think about it and ask for advice, you figured out how to apologize, and now you are getting along well again.

**Part 2: Growth Testimonials** *(10-12 mins)*

- You’ll now share some examples of how other people have faced failures and challenges and grown as a result.
  - In each of these stories, someone:
    - Faced a challenge or setback
    - Used effort and strategies to overcome the challenge
    - Learned or improved or grew as a result of the challenge
- **Ask volunteers to read the stories of Eunice Mwabe and Kago Kagichiri on page 3 of the booklet (“3. Growth Testimonials”).**
- **Lastly, quickly provide your own growth story (as a group leader)**
  - You will have prepared this story ahead of time, so make a note here of what your story is about.

***~40 mins left***

**Part 3: Assignment “Write your own growth story”** *(6-7 mins)*

- Tell the students that they will each write their own growth story
  - **Pass out “4. A Time You Grew” sheet**
  - Emphasize that students should include three things in their story:
    - The challenge they faced
    - How they used effort or strategies to deal with the challenge, and any setbacks they faced along the way
    - How they learned or improved or grew as a result of the challenge
  - Give them 5-6 mins to finish the sheet

**Part 4: Discussion about growth stories** *(5-6 mins)*

- **Lead a discussion about the growth stories.**
  - Sample questions:
    - Would some of you please share what you wrote?
    - What strategies did you use to overcome your challenge?
    - Did you face any failures along the way in your growth story?
    - How did it feel completing this activity?
  - During this discussion about personal growth stories, try to:
    - **Validate** and **restate** the three parts of participants’ stories.
      - Example: “Thank you so much for sharing. It seems like you were having trouble in physics (Part 1: Challenge), so you decided to talk with the teacher more and study more (Part 2: Effort/strategies), and now you love physics! (Part 3: Growth).
    - **Emphasize** the parts of the story that involve **effort** and **specific** **strategies**
      - Example: It sounds like the strategies you used were very helpful! You did more practice problems and you spoke with your teacher.
    - **Highlight** that growth does not happen because of “magic”
      - It requires effort, hard work, and strategies, and sometimes it’s not perfect.
    - **Highlight that often, you face failures along the way and you can learn from these failures**
      - Example: first, you tried doing more practice problems. But you didn’t improve immediately because there were some you couldn’t figure out on your own, so you reached out to a friend who helped you with the problems you didn’t understand.

**Part 5: Letter to a Friend** *(10 mins)*

- So far, we’ve learned about neuroplasticity and how our brains can grow, we’ve brainstormed some strategies that can help us, and we have talked about steps for solving problems. Now, we’re going to try to use what we learned in order to help others.
  - **Give students the “5. Letter to a Friend” sheet.**
- **Tell students to read the instructions aloud.**
- Let the students know that the letter will remain confidential. No one will see it except the research team.
- Ask if they have any questions about the prompt.

***~15 mins left***

**Part 6: Explain the Growth Take-Home Activity** *(2 mins)*

- **Pass out the “6. Growth Take-Home Activity Week 2” sheet**
- **Ask students to read the instructions aloud**
  - Identify a challenge you face during the next week
  - Write about how effort, strategies, and knowledge about growth to help you handle the challenge
  - Write about how you could grow as a result of the challenge
- Remind students that we will be checking to make sure they did the homework. You can only enter the raffle if you do the homework
- Ask if anyone has any questions
- Tell them you look forward to seeing them next week.

**Part 7: Midpoint Questionnaires** *(12 minutes)*

- Pass out the questionnaires
- Tell students to take about 10 minutes to fill them out
- Remind students before they fill out the measures:
  - Their responses will be kept private (no one except the study team will see them)
  - No one at the school will see their responses – the administration and teachers will not have access to them.
  - There are no right or wrong answers; they will not be graded
  - Please answer honestly
- Answer any questions that students have
- Collect the questionnaires.
- Ask if there are any questions about the HW assignment.
- Tell the students you look forward to seeing them at the same time, in the same place next week.

**Session 3 : Strategies for Growth**

- **Required Sheets:**
  - 7. What Helps You
  - 8. Effective Strategies List
  - 9. STEPS for Solving Life’s Problems
  - 10. Growth Take Home Activity Week 3
- **Session Overview:**
  - Part 1: Lead a discussion about the Growth Take Home Activity *(10 mins)*
  - Part 2: Introducing the Effective Strategies Discussion *(2-3 mins)*
  - Part 3: Discussion About Strategies Part 1: What Helps You? *(5 minutes)*
  - Part 4: Discussion About Strategies Part 2: Discussion *(10 minutes)*
  - Part 5: Solving Problems to Grow *(15 mins)*
  - Part 6: Explain the Problem Solving Growth Take-Home Activity *(2-3 mins)*

**Part 1: Lead a discussion about the Growth Take Home Activity** *(10 mins)*

- Sample questions:
  - - Does someone want to share what challenge they faced, and how they handled it?
    - What strategies or techniques did you use to handle the challenge?
    - What was rewarding about the activity?
    - What was challenging about the activity?
- During this discussion about the HW assignment, try to:
  - **Validate** and **restate** the three parts of participants’ homework
    - Example:
      - “Thank you so much for sharing. It seems like you were having trouble in physics (Part 1: Challenge), so you decided to talk with the teacher more and study more (Part 2: Effort/strategies), and now you think you will see improvement in physics! (Part 3: Growth).
  - **Emphasize** the parts of the story that involve **effort** and **specific** **strategies**
    - Example:
      - It sounds like the strategies you used were very helpful! You did more practice problems and you spoke with your teacher.
  - **Highlight** that growth does not happen because of “magic”.
    - It requires effort, hard work, and strategies, and sometimes it’s not perfect.
  - **Highlight that often, you face failures along the way and you can learn from these failures**
    - Example:
      - First, you tried doing more practice problems. But you didn’t improve immediately because there were some you couldn’t figure out on your own, so you reached out to a friend who helped you with the problems you didn’t understand.

**Part 2: Introducing the Effective Strategies Discussion** *(2-3 mins)*

- Now that we’ve learned that we can grow and improve, we’re going to talk about strategies we can use to help us grow.
- Remember, growth is **not** magic; growth can take time and be difficult, and it requires effort and strategies.
- Today, we’re going to create a list of strategies we use to improve our wellbeing, academics, and more.
- Remember, no single strategy is perfect for each person or every situation; the goal of this discussion is to find a few strategies that work for you.
- Different people prefer different strategies, and different strategies are useful in different situations. It’s OK if you don’t think every strategy on the list will be helpful—hopefully, each of you will have a few strategies you like on the list
- Does anyone have questions?

***~33 mins left***

**Part 3: Discussion About Strategies Part 1: What Helps You?** *(5 minutes)*

- Tell the students that you’re going to ask them to think about what strategies they use to grow and overcome challenges.
- **Pass out the “7. What helps you?” sheet.**
- **Ask the students to read the instructions aloud.**
- Then, ask the students to write down a few strategies on their own on the sheet. (*4-5 mins)*
  - These strategies can be things that they do when they feel upset, when they have a conflict with another person, or things that they do to improve their academics

**Part 4: Discussion About Strategies Part 2: Discussion** *(10 minutes)*

- **Next, lead the discussion by asking people to share what they wrote down.**
- Sample Questions for the discussion:
  - When you feel upset, what types of things do you do to feel better?
  - If you’re worried about something, what do you to relax?
  - If you’re feeling sad, what might you do to cheer yourself up?
  - When you’re worried about an assignment or an exam, what strategies do you use?
  - If you have a conflict with someone else, what might you do to improve the situation?
- During this discussion, try to:
  - **Ask follow-up questions** to get students to be as **specific** as possible.
    - Example:
      - So when you’re upset, it helps when you talk with your friends. What do you usually say to your friends? Do you talk about the thing that’s bothering you, or something else? How do you decide which friends to talk to?
  - **Validate** and **relate to** some of the strategies. If possible, make their strategies more specific or occasionally suggest extra strategies.
  - **Refer** to the “What helps you” sheet if you want to add some examples to the list.
    - Example:
      - “Yeah, reaching out to friends can be really helpful. Sometimes, I even ask them to go on a walk with me. That way, I get to talk with them and I also move around a bit.”
    - Example:
      - “Yeah, writing about the problem can be really nice. One of my friends told me that she thinks about how someone she admires would handle the problem.”
- **Pass out the “8. Effective Strategies List” for the students to look at and learn some other helpful strategies.**

***~18 mins left***

**Part 5: Solving Problems to Grow** *(15 mins)*

- - Now we are going to talk more about how you can grow.
  - We will discuss how to identify areas for growth and use some of the strategies we discussed before to solve problems in your lives and help you grow.
- **Pass out “9. STEPS for Solving Life’s Problems” Sheets**
- **Ask: Will each of you read one of the steps from the sheet?**
- As they read, briefly explain each of the STEPS:
  - S:
    - Say What the Problem Is:
      - Figure out exactly what the problem is you want to solve
  - T:
    - Think of Solutions:
      - Make a list of three or more possible solutions even if you don’t think you’ll use all of them
  - E:
    - Examine Each One:
      - Positives and Negatives
        - Think about the things you like and don’t like about each solution you thought of
  - P:
    - Pick One:
      - Pick the best solution for you
  - S:
    - See if it Works:
      - Consider what might go wrong when trying that solution and how you could handle these issues.
      - This may sometimes mean selecting another solution
  - Finally:
    - Try the solution:
      - Do the thing you chose to do, and evaluate how it went, then go through the steps again if you need to
- **Group leaders should share a simple problem they are facing (can be made up)**
  - Example:
    - You’ve been arguing with your sister over doing enough chores.
    - Ask the students to walk through the steps to solve the problem with you *(5 mins)*
- Tell them they will all fill out the sheet later at home to practice more problem solving

**Part 6: Explain the Problem Solving Growth Take-Home Activity** *(2-3 mins)*

- Pass out the “**10. Growth Take Home Activity Week 3**” worksheet
- Explain the HW assignment.
  - Ask students think of a problem of their own that they could use problem solving to help solve; it should be something that is bothering them or has been on their mind
  - Remind everyone to try and pick a problem that they have some control over and that isn’t too huge. For example, it is too hard to create world peace in a week!
- **Fill out the sheet for their own problem for HW**
- Try out the solution they decide on this week and see how it goes

**Session 4: Growth in the Future**

- **Required sheets**
  - 11. Setting a Goal for Growth
- **Session Overview**
  - Part 1: Discussion About Problem Solving Take-Home Assignment *(10 mins)*
  - Part 2: Discussing Specific Goals (*15 mins)*
  - Part 3: Conclusion *(15 mins)*
  - Part 4: Endpoint measures (including feedback) *(20 mins)*

**Part 1: Discussion About Problem Solving Take-Home Assignment** *(10 mins)*

- What problem did you choose to try and solve this past week?
- How did you go about identifying the problem?
- What solution did you pick, and why did you pick the one you did?
- How did it go when you tried to act on your solution?
- Did you encounter any obstacles? If so, how could you pick a different solution or get around them in the future?
- During this discussion about the HW assignment, try to:
  - **Validate** the ways in which students used the problem-solving framework correctly
    - Example:
      - “Thank you for telling us about the problem you worked on, which was getting in trouble for talking during class. It sounds like you considered some possible solutions, and when you tried the first and it didn’t work, you tried another which helped a lot more!
  - **Emphasize** the parts of the story that involve **effort, learning from challenges,** and **growth**
    - Example:
      - It sounds like you did a great job of learning from the challenges you faced when you first tried to solve this problem. The challenge you encountered helped you come up with an even better strategy for solving your problem!

**Part 2: Discussing Specific Goals** (*15 mins)*

- Say: break into groups of two by turning to the person next to you
- **Hand out the “11. Setting a Goal for Growth” worksheet**
  - Tell the students they should discuss their answers with their partner and fill in the sheets together.
  - The three parts of the activity are:
    - Select a specific problem, challenge, or area for growth – try to make it something specific, and something you have some control over
    - Write down why you would like to grow in that area
    - Write down how you think you might grow in that area (list some specific strategies or ways of solving the problem)

***~35 mins left***

**Part 3: Conclusion** *(15 mins)*

- Lead a wrap-up discussion about growth mindset.  This discussion will start with each student going around and sharing one way in which they will use what they learned in their own life.
  - **Ask:** We have talked a lot about growth, neuroplasticity, and strategies for growth like problem solving. Now, we will all go around and share one specific why we will use what we learned in the future in our own lives. Who would like to start?
    - Let each student share what they will use in the future.
    - Validate their responses.
- Then, if you have time, lead a broader discussion about how the students will use what they learned.
  - Sample questions:
    - How can you use the ideas we’ve learned about in your lives?
    - Are there any strategies you want to use this week?
    - How can the stuff we talked about help you in your academics?
    - How can the stuff we learned about help you in your relationships?
    - How can the stuff we learned about help you overcome challenges?
- **Throughout this discussion:**
  - **EMPHASIZE** how **you can grow in many different aspects of life**, including happiness, friendships and family relationships, and intelligence.
  - **Highlight** how **growth requires effort and strategies,** and how often **growth involves setbacks** along the way.

***~20 mins left***

**Part 4: Endpoint measures (including feedback)** *(20 mins)*

- **Pass out the questionnaires**
- Tell students to take about 20 minutes to fill them out
- Remind students before they fill out the document:
  - Their responses will be kept private (no one except the Shamiri team will see them)
  - No one at the school will see their responses – the administration and teachers will not have access to them.
  - There are no right or wrong answers; they will not be graded
  - You should answer as honestly as possible.
- Answer any questions that students have.
- Explain that this will be the last part of the program.
- Tell your students that you enjoyed working with them and thank them for being good students.
- When the students are finished, collect their questionnaires.
