# Supplementary material for: Testing the effects of the Shamiri Intervention and its components on anxiety, depression, wellbeing, and academic functioning in Kenyan adolescents: study protocol for a five-arm randomized controlled trial
Source: Trials. 2021 Nov 22;22:829. doi: 10.1186/s13063-021-05736-1 (PMC8607059; doi:10.1186/s13063-021-05736-1)
Supplement: Supplementary file 7 — Additional file 7. . [file 13063_2021_5736_MOESM7_ESM.docx]

**Shamiri Gratitude-Only Program Protocol for Group Leaders (Lay – Providers)**

**Follow the protocol:** Don’t add anything or omit anything (unless you are running short on time, in which case you can cut discussions short by calling on fewer students.)

**Watch the time:** Look at the time allotted for each section, and check your watch, clock, or phone to make sure you don’t run overtime.

**Handling incorrect responses:** Everyone is unique, and we will respect those differences. However, if someone says something clearly incorrect you may: ask others in the group what they think about it, point out the issues with it in a lighthearted way, gently correct the student’s misunderstanding yourself, or offer to speak with the student more after the session if they are still confused or don’t agree with you.

**Handling risk:** Refer to your risk protocol for details. If you are worried that a participant may harm her/himself or others, **speak to your supervisor** as soon as you can (usually right after the session).

**Gratitude Protocol**

**Session 1:**

- Required Sheets:
  - 1. Gratitude Article
  - 2. Three Good Things
- Session Overview:
  - Part 1: Questionnaires *(5-10 min)*
  - Part 2: Icebreaker *(10-15 min)*
  - Part 3: Introduction *(5 min)*
  - Part 4: Introduce rules and expectations for the group *(3-4 min)*
  - Part 5: Explain concept of gratitude *(3 min)*
  - Part 6: Gratitude article and video *(6-8 min)*
  - Part 7: Group discussion *(10 min)*
  - Part 8: Three Good Things HW *(3-4 min)*

**Part 1: Questionnaires** *(5-10 min)*

- Pass out the questionnaires
- Hand out pens and notebooks to each student.
- Tell students to take about 5 minutes to do the questionnaires
- Remind students before they start the questionnaires:
  - The questionnaires will ask about how you’ve been doing (socially academically, and in terms of wellness)
  - Responses will be kept private (no one except the study team will see them)
  - No one at the school will see your responses – the administration and teachers will not have access to them
  - There are no right or wrong answers; you will not be graded
  - You should answer as honestly as possible
- Answer any questions that students have
- Collect the questionnaires

**Part 2: Icebreaker** *(10-15 min)*

- Introduce yourself and ask everyone’s name (keep this short!)
  - Icebreaker (pick ONE ice breaker activity you think your group will like)
    - Examples:
      - Two truths and a lie (everyone says two true things and one lie about themselves; other group members guess which one is the lie)
      - Guess who wrote it (each person should write down one hobby they have and why they like it; Read out the hobbies one by one and have other group members guess who wrote down each hobby)
      - Rosebud/thorn (have everyone go around and say a highlight from their week, a low point of their week, and something they are looking forward to)

***~40 min left***

**Part 3: Introduction** *(5 min)*

- This program is important because it is designed to:
  - - Help students achieve their goals, feel happier, do better in school, and improve their lives
    - Help them learn how to handle problems and improve their overall wellbeing
- The program is designed to improve wellness and academic performance
  - Uses research from **Harvard University** and **Stanford University**
  - This research has **helped students right here in Kenya, and in America and Europe**
  - **Results from the last several years showed that activities like the ones we are going to do helped students from Kenya to feel happier and get better grades**
- Students who participated in this study last year said that it helped them to:
  - Build better social relationships, do better in school, feel happier, have more hope and more skills for the future, learn to better handle challenges, and figure out what is most important to them
- Throughout this program, we will be talking about ways in which each of you can improve your life.
  - So, as we learn over the next few weeks, try to think about how what you learn can apply to you
  - For example, maybe it can help you improve your family relationships or friendships, achieve your goals, or feel better

**Part 4: Introduce rules and expectations for the group** *(3-4 min)*

- Ask: **How do you want people to act in the groups? What rules do you want to set?** (Allow 3-4 students to speak, then say other rules; make sure to include the rules below; you may also have other rules that students come up with)
  - **Be respectful of others**
  - Don’t talk over anyone
  - Participate as much as you would like
    - Be open and honest
    - Anything you want to say or not say is okay
  - Carry your pen and notebook to the sessions.
  - **We want to hear from all of you – please participate if you feel comfortable**
  - **Confidentiality: Do NOT** **share anyone’s personal information**. *THIS IS IMPORTANT!!!!*
    - Explain how you wouldn’t like it if your secrets were shared, so they shouldn’t share anyone else’s information
- Also, it is important that you not tell others at the school about what you learn, because we are doing a scientific study of this program, and if you tell others what you learned, we might not be able to tell how well the program works.
- **We won’t share anything that you say in the groups with anyone else; It will all stay private from everyone else at school. The only situation in which we would have to tell someone else is if you tell us you’re thinking of seriously harming yourself or someone else, in which case we will have to tell someone from the study team, or possibly from the school. In all other cases, everything will stay private!**
- If you break the rules, we will:
  - Talk with you one-on-one
  - Not allow you to enter the t-shirt raffle
  - If you continue to break the rules, we will remove you from the group and talk with your principal
- Pause and ask if anyone has questions
- Then, ask them to all: Can you commit to following the rules?
  - They should all say “yes” to this.

**Part 5: Explain concept of gratitude** *(3 min)*

- Today, we’re going to be talking about gratitude.
  - Parents or teachers might have told you that you should be more grateful before, but that’s not what we are trying to do here
  - It’s normal and okay to sometimes not feel grateful!
  - But we can approach this in a way that allows you to feel grateful for the things you want to feel grateful for, and that allows you to benefit from gratitude
- Gratitude is noticing things that you like or that are going well, and appreciating those things. This week, we’re going to identify good things in our lives that we are thankful for and show our gratitude.
- People can be grateful for literally anyone or anything that’s impacting their life positively. (Give a personal example.)
- Showing our gratitude through words is very important, both for our own happiness, and for the happiness of the people around us
  - Thinking of specific things we are grateful for also improves our sleep, relationships, and overall well-being
- It’s not always easy to feel grateful. However, through practice, gratitude gets easier and can help you more over time

***~25 min left***

**Part 6: Gratitude article and video** *(6-8 min)*

- Pass out sheet: **1. Gratitude Article** *(3-4 min)*
  - Ask for a volunteer to read each paragraph
- Show the two-minute video on gratitude *(2-3 min)*
  - Briefly summarize the article and video in your own words *(1-2 min)* In your explanation, mention that:
    - Gratitude is similar to being thankful
    - Gratitude involves noticing and appreciating good things
    - You can be grateful for many different things
    - Gratitude can help you live the life you want to live and feel better about your life and yourself

**Part 7: Group discussion** *(10 min)*

- **Lead a discussion about the article and the video**
  - Sample questions:
    - Can you describe gratitude in your own words?
    - What/Who in your life are you grateful for? Why? Every participant can share briefly if they are comfortable.
    - What has happened in the past few days that has made you feel happy?
    - What good things have happened to you recently?
    - Who has done something nice for you recently?
- During this discussion, try to:
  - **Validate** and **restate** the parts of students’ answers that accurately describe gratitude
  - **Emphasize** that you can be grateful for many different things
  - **Validate** that feeling gratitude can require effort and practice

**Part 8: Three Good Things HW** *(3-4 min)*

- **Make sure all students have the sheet: “2. Three Good Things”**
- At the end of each day, write Three Good Things or People for which you are grateful
- For each good thing, answer ONE of the questions in the second column. You don’t have to answer the same question for each thing
- **Ask the students to go around and read the activity instructions**
- Ask one or two students to share an example of something they might write down on the sheet

**Session 2:**

- **Required Sheets:**
  - 3. Gratitude Testimonials
  - 4. Gratitude Story Prompt
  - 5. Making Gratitude a Habit
  - 6. Three Good Things with a Twist
- **Session Overview:**
  - Part 1: Review and Discussion about HW *(10 min)*
  - Part 2: Gratitude Testimonials *(10 min)*
  - Part 3: Your own Gratitude Story *(10 min)*
  - Part 4: Expressing your Gratitude Pair-Share and Discussion *(10 min)*
  - Part 5: Habit Formation/Implementation Intentions *(5 min)*
  - Part 6: Take-Home Activity *(3 min)*
  - Part 7: Midpoint Questionnaires *(12-15 min)*

**Part 1: Review and Discussion about HW** *(10 min)*

- What did you feel grateful for over the past week?
- What/Who in your life are you grateful for? Why are you grateful for these things/people?
- What has happened in the past few days that has made you feel happy? What good things have happened to you recently?
- How could you make these good things happen more often?
- How did you express your gratitude, if you did?

**Part 2: Gratitude Testimonials** *(10 min)*

- **Pass out the sheet: “3. Gratitude Testimonials”**
- Explain that you’ll now be providing some examples of how people have benefitted from gratitude.
  - Explain that in each of these stories, someone:
    - Was struggling in some way
    - Used gratitude to help themselves feel better
    - Found that, because of gratitude, they began to feel better and their life improved
    - Pass out the “Gratitude Testimonials” Sheet
    - Ask a student to read each of the testimonials aloud

***~40 min left***

**Part 3: Your own Gratitude Story** *(10 min)*

- **Pass out the sheet: “4. Gratitude Story Prompt”**
- Ask the students to:
  - Write about a time when you felt really grateful, what you felt grateful for, and how it affected your life to feel grateful
  - Why were you grateful? Was there anything you did when you were grateful to express your gratitude?

**Part 4: Expressing your Gratitude Pair-Share and Discussion** *(10 min)*

- Ask the students to break into groups of two with the person next to them
- In this small group, the students should talk about
  - Ways they usually express gratitude to other people
  - Ways they think they could express gratitude in the future
- Bring the whole group back together to discuss ways of expressing gratitude. Here are some questions you can ask:
  - How do you usually express gratitude?
  - What are some ways of expressing gratitude that you would like to use more in the future?
  - Do you have different ways of expressing gratitude to different people? For example, friends, family, and teachers?
  - Can you think of any people you’d especially like to express gratitude to? How would you like to express it?

***~20 min left***

**Part 5: Habit Formation/Implementation Intentions** *(5 min)*

- **Pass out sheet: “5. Making Gratitude a Habit”**
  - Ask students to read it aloud
- Briefly explain what a habit is: a regular thing that you do. Ex: brushing teeth, taking a shower, eating breakfast
- Adding writing in a gratitude journal to an existing habit such as brushing your teeth, taking a shower, or eating can help you remember to practice it every day
  - Remember that if you still forget some days, you can always start up again!
- Ask the students if they have any questions

**Part 6: Take-Home Activity** *(3 min)*

- **Pass out the sheet:** **“6. Three Good Things with a Twist”**
- **Explain** that this is the same exercise they did last week; just with a twist!
- Every day write down one good thing you feel grateful or thankful for. It could be anything -- an object, a person, or an experience
- Write about it at the same time/with the same trigger (e.g., after you brush your teeth)
- It doesn’t have to be very big or super exciting, it just has to be something you liked or that made you feel happy
- Then write about why that thing was important to you and how you might be able to make that thing happen more often
- There are no right or wrong answers

**Part 7: Midpoint Questionnaires** *(12-15 min)*

- Pass out the questionnaires
- Tell students to take about 10 minutes to fill them out
- Remind students before they fill out the measures:
  - Their responses will be kept private (no one except the study team will see them)
  - No one at the school will see their responses – the administration and teachers will not have access to them
  - There are no right or wrong answers; they will not be graded
  - Please answer honestly
- Answer any questions that students have

**Session 3:**

- **Required Sheets:**
  - 7. What am I Grateful for about Myself?
  - 8. Gratitude Letter
  - 9. Saying Thank You
- **Session Overview:**
  - Part 1: Discussion about Three Good Things with a Twist HW *(10 min)*
  - Part 2: What am I Grateful for about Myself? *(10 min)*
  - Part 3: Gratitude Letter *(15 min)*
  - Part 4: Discussion about the gratitude letter *(10 min)*
  - Part 5: Homework – Saying Thank You *(4 min)*

**Part 1: Discussion about Three Good Things with a Twist HW** *(10 min)*

- Sample questions:
  - How did the activity go for you?
  - Does someone want to share one thing they were grateful for and how they reflected on it?
  - Do you want to do something like this in the future?
  - What are some ways that we can continue to feel and express gratitude?
  - Did you find that doing this at the same time every day worked for you, or did it not? Did it help you remember to practice gratitude?
- During this discussion, try to:
  - Validate the things that students share.
  - Restate the reflections the students offer. Emphasize reasons why the “good thing” is important to students and strategies they can use to make the good thing happen more in the future.
    - Example: “Thank you so much for sharing. It seems like you really enjoyed your conversation with your friend. It sounds like if you talk to that friend more often, you’ll be able to have even more of these conversations that make you feel happy.”
  - Ask other students if they were grateful for any of the same things.
    - Example: “Did anyone else write about being grateful for a friend this week?”

**Part 2: What am I Grateful for about Myself?** *(10 min)*

- **Pass out sheet: “7. What am I Grateful for about Myself?”**
- In this activity, you will get a chance to think about things about yourself that you like or are grateful for
- We will ask you to think of one thing about yourself that you’re thankful for
- You’ll then get a chance to reflect on why you are grateful for this thing about yourself
- You won’t have to share your answers with anyone else for this activity
- **Ask the students to go around and read the activity instructions**
- **Do not discuss this activity as a group**

***~40 min left***

**Part 3: Gratitude Letter** *(15-20 min)*

- **Pass out the sheet: “8. Gratitude Letter”**
- Explain to the students that they should think of one person to write a letter to who:
  - Did something for them or helped them in some way
  - They haven’t properly thanked.
- In that letter, you should describe what the person did for you, why you’re grateful, and how they changed your life
- **Explain:** They can give the letter to that person later, or they can keep the letter private, or give it to the group leader for the study team. We recommend they give it to the person they wrote it about, but they don’t have to.
- For homework, we’ll recommend they give the letter to the person, but they can opt out if it’s not possible to give the letter to the person they wrote it for.

**Part 4: Discussion about the gratitude letter** *(10-15 min)*

- Generally, what was it like completing this exercise? How did it feel to write a letter to someone you’re grateful to?
- What types of changes did you notice in yourself while completing the exercise?
- What was helpful about the exercise?
- Is this something that you want to keep doing? Are there any other people in your life that you might want to write a letter for?

***~15 min left***

**Part 5: Homework – Saying Thank You** *(4 min)*

- **Pass out the sheet: “9. Saying Thank You”**
- Explain that the homework will be to practice expressing our gratitude to others
- Research shows that expressing thanks doesn’t just make others feel great, it also makes *you* feel happier and have better relationships!
- For this homework, we will ask you to share your gratitude letter with the person you wrote it for
- Or, you can say thank you to someone else for helping you or making you happy in another way
- Or, you can do both!

**Session 4:**

- **Required Sheets:**
  - 10. Savoring Activity
- **Session Overview:**
  - Part 1: Review Saying Thank You HW *(10 min)*
  - Part 2: Savoring Activity *(10-12 min)*
  - Part 3: Conclusion *(10 min)*
  - Part 4: Closing Group Share Reflection *(5-7 min)*
  - Part 5: Endpoint measures (including feedback) *(20-25 min)*

**Part 1: Review Saying Thank You HW** *(10 min)*

- **Lead a discussion about the HW.**
- **Sample Questions:**
  - How did you express your gratitude to someone else?
  - How did it go for you?
  - How did you feel about expressing your gratitude?
  - Did any problems come up, or would you do it differently in any way next time?
  - How could you continue to express gratitude to others in the future?
  - Is there anyone else you’d like to thank?
  - Is there anything else you’d want to do for people that they might appreciate?

**Part 2: Savoring Activity** *(10-12 min)*

- **Pass out the sheet: “10. Savoring Activity”**
  - Savoring means really enjoying something
  - For example, think about your favorite treat, and how much you enjoy it when you really get to focus on enjoying it
  - Mention how sometimes, things can seem so much more enjoyable if we really take time and make effort to savor them. For example, think about eating a meal in a hurry in comparison to really savoring a meal.
  - You can savor something as it is happening, or after it happens.
- Now, please reflect on something you really enjoy.
- In the activity, you’ll also think about how you can savor this thing more often and more fully.

***~40 min left***

**Part 3: Conclusion** *(10 min)*

- Lead a wrap-up discussion about gratitude. This discussion does **not** have to focus on the most recent activity.
  - Sample questions:
    - How can you use the ideas we’ve learned about in your lives?
    - Are there any strategies you want to use this week?
    - How can the stuff we talked about help you in your academics?
    - How can the stuff we learned about help you in your relationships?
    - How can the stuff we learned about help you overcome challenges?
- **Emphasize how** **you can experience more gratitude and spread more gratitude to those with whom you’re close**. Feeling grateful can help you in a lot of ways. You can always work to be more grateful, even if your life will never be perfect.

**Part 4: Closing Group Share Reflection** *(5-7 min)*

- Ask everyone to go around and each say one thing they will use in the future or that they learned from the groups as a whole, not just the last session.

***~25 min left***

**Part 5: Endpoint measures (including feedback)** *(20-25 min)*

- **Pass out the questionnaires**
- Tell students to take about 20 minutes to fill them out
- Remind students before they fill out the document:
  - Their responses will be kept private (no one except the study team will see them)
  - No one at the school will see their responses – the administration and teachers will not have access to them
  - There are no right or wrong answers; they will not be graded
  - You should answer as honestly as possible
- Answer any questions that students have
- Explain that this will be the last part of the program
- Tell your students that you enjoyed working with them and thank them for being good students
- When the students are finished, collect their questionnaires

----------------------------------------------------------------------------------------------------------------------------
