# Supplementary material for: Testing the effects of the Shamiri Intervention and its components on anxiety, depression, wellbeing, and academic functioning in Kenyan adolescents: study protocol for a five-arm randomized controlled trial
Source: Trials. 2021 Nov 22;22:829. doi: 10.1186/s13063-021-05736-1 (PMC8607059; doi:10.1186/s13063-021-05736-1)
Supplement: Supplementary file 8 — Additional file 8. . [file 13063_2021_5736_MOESM8_ESM.docx]

**Shamiri Values-Only Program Protocol for Group Leaders (Lay – providers)**

**Follow the protocol:** Don’t add anything or omit anything (unless you are running short on time, in which case you can cut discussions short by calling on fewer students.)

**Watch the time:** Look at the time allotted for each section, and check your watch, clock, or phone to make sure you don’t run overtime.

**Handling incorrect responses:** Everyone is unique, and we will respect those differences. However, if someone says something clearly incorrect you may: ask others in the group what they think about it, point out the issues with it in a lighthearted way, gently correct the student’s misunderstanding yourself, or offer to speak with the student more after the session if they are still confused or don’t agree with you.

**Handling risk:** Refer to your risk protocol for details. If you are worried that a participant may harm her/himself or others, speak to your supervisor as soon as you can (usually right after the session).

**Values Protocol**

**Session 1:**

- **Required sheets:**
  - 1. What are Values and Why do they Matter?
  - 2. What Matters to Me
  - 3. Noticing Values in Real Life Week 1 HW
- **Session overview:**
  - Part 1: Questionnaires *(10-15 min)*
  - Part 2: Icebreaker *(10-15 min)*
  - Part 3: Introduction *(5 min)*
  - Part 4: Introduce rules and expectations for the group *(3-4 min)*
  - Part 5: Explain concept of values (*4 min)*
  - Part 6: Values article and video *(8-10 min)*
  - Part 7: Group discussion *(8 min)*
  - Part 8: HW assignment – Noticing values in real life

**Part 1: Questionnaires** *(10-15 min)*

- Pass out the questionnaires
- Hand out pens and notebooks to each student.
- Tell students to take about 5 minutes to do the questionnaires
- Remind students before they start the questionnaires:
  - The questionnaires will ask about how you’ve been doing (socially academically, and in terms of wellness).
  - Responses will be kept private (no one except the study team will see them).
  - No one at the school will see your responses – the administration and teachers will not have access to them.
  - There are no right or wrong answers; you will not be graded.
  - You should answer as honestly as possible.
- Answer any questions that students have
- Collect the questionnaires

**Part 2: Icebreaker** *(10-15 min)*

- Introduce yourself and ask everyone’s name (keep this short!)
  - Icebreaker (pick ONE ice breaker activity you think your group will like)
    - Examples:
      - Two truths and a lie (everyone says two true things and one lie about themselves; other group members guess which one is the lie)
      - Guess who wrote it (each person should write down one hobby they have and why they like it; Read out the hobbies one by one and have other group members guess who wrote down each hobby)
      - Rosebud/thorn (have everyone go around and say a highlight from their week, a low point of their week, and something they are looking forward to)

***~40 mins left***

**Part 3: Introduction** *(5 min)*

- This program is important because it is designed to:
  - - Help students achieve their goals, feel happier, do better in school, and improve their lives.
    - Help them learn how to handle problems and improve their overall wellbeing.
- The program is designed to improve wellness and academic performance
  - Uses research from **Harvard University** and **Stanford University**
  - This research has **helped students right here in Kenya, and in America and Europe**
  - **Results from the last several years showed that activities like the ones we are going to do helped students from Kenya to feel happier and get better grades**
- Students who participated in this study last year said that it helped them to:
  - Build better social relationships, do better in school, feel happier, have more hope and more skills for the future, learn to better handle challenges, and figure out what is most important to them
- Throughout this program, we will be talking about ways in which each of you can improve your life.
  - So, as we learn over the next few weeks, try to think about how what you learn can apply to you.
  - For example, maybe it can help you improve your family relationships or friendships, achieve your goals, or feel better.

**Part 4: Introduce rules and expectations for the group** *(3-4 min)*

- Ask: **How do you want people to act in the groups? What rules do you want to set?** (Allow 3-4 students to speak, then say other rules; make sure to include the rules below; you may also have other rules that students come up with)
  - **Be respectful of others**
  - Don’t talk over anyone
  - Participate as much as you would like
    - Be open and honest
    - Anything you want to say or not say is okay
  - Carry your pen and notebook to the sessions.
  - **We want to hear from all of you – please participate if you feel comfortable**
  - **Confidentiality: Do NOT** **share anyone’s personal information**. *THIS IS IMPORTANT!!!!*
    - Explain how you wouldn’t like it if your secrets were shared, so they shouldn’t share anyone else’s information
- Also, it is important that you not tell others at the school about what you learn, because we are doing a scientific study of this program, and if you tell others what you learned, we might not be able to tell how well the program works.
- **We won’t share anything that you say in the groups with anyone else; It will all stay private from everyone else at school. The only situation in which we would have to tell someone else is if you tell us you’re thinking of seriously harming yourself or someone else, in which case we will have to tell someone from the study team, or possibly from the school. In all other cases, everything will stay private!**
- If you break the rules, we will:
  - Talk with you one-on-one
  - Not allow you to enter the t-shirt raffle
  - If you continue to break the rules, we will remove you from the group and talk with your principal
- **Pause and ask if anyone has questions**
- **Then, ask them to all: Can you commit to following the rules?**
  - **They should all say “yes” to this.**

***~25 mins left***

**Part 5: Explain concept of values (***4 min)*

- Describe what values are
  - Example: “Values are things that are important to you; often, your values affect the ways you want to live your life. They are things or actions that you think are good and that you admire. They might mean a lot to you.”
- Note that values are similar to virtues, which you may have learned about before.
- Give some examples of possible values, using your own values
  - Example: “For example, one of my values is kindness. I want to be kind, especially to my friends. Helpfulness is also one of my values. I want to be helpful, even to strangers. I also value being a strong student and being a helpful group leader. Everyone has different values, different things that are important to them.”
- Add that it’s okay if you don’t yet know your values. We will be doing some exercises to help you identify them.
- Add that it is normal for your values to change over time, and you might develop new values in the future.

**Part 6: Values article and video** *(8-10 min)*

- Pass out the article on values “**1. What are Values** **and Why do they Matter?**” *(4-5 minutes).*
  - Ask for a volunteer to read each paragraph
- When they finish, ask for another volunteer to read the second paragraph. Repeat this until the students finish reading the article.
- Show the two minute video on values. (2-3 minutes)
  - Briefly summarize the article and video in your own words. (1-2 mins). In your explanation, mention that:
    - Values are principles, ideals, and life goals that are important to you
    - They inform how you want to live your life
    - Understanding and acting on your values can help you live the life you want to live and feel better about yourself.
    - Give an example of a value and its importance

***~10 mins left***

**Part 7: Group discussion** *(8 min)*

- **Lead a discussion about the article and the video**
- Can you describe values in your own words?
- Can you give some examples of possible values?
  - Pass out the **“2. What Matters to Me”** worksheet with examples of values.
  - Explain that this sheet gives more examples of common values
- Do you have any questions about values?
- During this discussion, try to:
  - **Validate** and **restate** the parts of students’ answers that accurately describe values
  - **Emphasize** that you can have many different values over time
  - **Validate** that values can help guide your life choices

**Part 8: HW assignment – Noticing values in real life**

- Worksheet **“3. Noticing Values in Real Life Week 1 HW”**
- This week, notice a time when you or someone else uses their values to make a decision, handle a challenge, or in some other way change what they do or say for the better.
- Make sure it’s something you are proud of or admire.
- Write about what value they display
- Why you admired it
- And anything you could learn from what you saw or did

**Session 2:**

- **Required sheets:**
  - 4. Testimonial Sheet
  - 2. What Matters To Me
  - 5. What’s Important to Me
  - 6. Using Values in Your Life HW Week 2
- **Session overview:**
  - Part 1: Brief Discussion of the HW *(5 min)*
  - Part 2: Stories of Role Models and Their Values *(10 min)*
  - Part 3: Group Leader Testimonial of Value Affirmations *(3-4 min)*
  - Part 4: Importance of Values Discussion *(8 min)*
  - Part 5: Introducing the Writing Activity: Past Experiences of Our Own Life *(4 min)*
  - Part 6: Value Affirmations Writing Prompt *(10 min)*
  - Part 7: HW for the Week *(3 min)*
  - Part 8: Midpoint Questionnaires *(12-15 min)*

**Part 1: Brief Discussion of the HW** *(5 min)*

- What did you write about?
- Did you write about a value you used? If so, what value and how did you use it?
- Did you write about something someone else did or said? If so, what value and how did they use it?
- What did you learn from this activity? How might it affect your own beliefs or life?

**Part 2: Stories of Role Models and Their Values** *(10 min)*

- **Pass out “4. Testimonial Sheet”**
- Explain that you’ll now be providing some examples of how people have benefitted from values and how values have helped them overcome challenges.
  - Each of these stories describes how someone:
    - Had a value that was especially important to them
    - That value guided their life and their decisions
    - Helped them to have a better life, maybe by being successful at their goals and by helping make them happy
- **Ask volunteers to read the stories.**

***~45 min left***

**Part 3: Group Leader Testimonial of Value Affirmations** *(3-4 min)*

- - **Group leaders present their own story of a role model who really showed an admirable value.** They emphasize three key elements in this story *(2-3 min):*
- Which key value the person chose and lived by
- How this value guided their life and decisions
- And how the value allowed them to be successful AND live a happy life.

**Part 4: Importance of Values Discussion** *(8 min)*

- Ask the students to break into groups of two with the person next to them
- **Discuss in the small groups** any other examples of a role model whose values helped them be successful AND achieve happiness in life *(3 mins)*
  - - **Discuss as a full group:**
      - Would any of you share the role models you discussed with your partner? *(4 mins total)*
    - **Reinforce** the key elements of each story
      - For example:
        - Selecting important personal values
        - Using it to help you decide how to live your life
        - Values helping with big and small decisions
        - Becoming more successful and happier as a result of identifying and using your values

***~20 min left***

**Part 5: Introducing the Writing Activity: Past Experiences of Our Own Life** *(4 min)*

- **Hand out “2. What Matters to Me” sheet with a list of values.**
  - - **Explain**: The list of values we handed out is not complete, and that students can add other values if something is missing that is important to them
- **You will have 3 mins to choose and circle the 3-5 values that are most important to you and your life right now.**

**Part 6: Value Affirmations Writing Prompt** *(10 min)*

- **Pass out the “5. What’s Important to Me” Sheet**
  - Group leaders explain writing task to students: students have 15 mins to choose **one** of their three values and write about a time when they really demonstrated that value.
  - **Ask students to go around and read the instructions aloud**
  - Remind them:
    - They can use their values to inform their decisions and to shape their futures
    - So, in addition to reflecting on how their vlaues have affected their life in the past, they will be asked to think about how values could inform their future.
  - Say: You should not worry about grammar or writing quality, because it is not going to be graded.
  - You can discuss students answers but ONLY if you have time.

**Part 7: HW for the Week** *(3 min)*

- Pass out “**6. Using Values in Your Life HW Week 2**” HW Sheet
- Ask students to keep their top value in mind as they face challenges or make decisions this week
- Everyone should identify one challenge or decision during the week and try and consider their top value in how they should deal with that challenge or make that decision.
- Write down the challenge or decision, and how your value might affect how you respond to it. Write down what you actually did or plan to do to deal with the challenge or decision.

***~15 mins left***

**Part 8: Midpoint Questionnaires** *(12-15 min)*

- Pass out the questionnaires
- Tell students to take about 10 minutes to fill them out
- Remind students before they fill out the measures:
  - Their responses will be kept private (no one except the study team will see them)
  - No one at the school will see their responses – the administration and teachers will not have access to them.
  - There are no right or wrong answers; they will not be graded
  - Please answer honestly
- Answer any questions that students have

**Session 3:**

- **Required sheets:**
  - 7. Making My Plans for Now and the Future
  - 8. Letter to Someone You Admire
- **Session overview:**
  - Part 1: HW Review *(5-10 min)*
  - Part 2: Making Plans Based in Values Activity *(10 min)*
  - Part 3: Discussion of Future Goals in the Short Term *(10 min)*
  - Part 4: Letter to Someone You Admire *(10 min)*
  - Part 5: Homework *(5 min)*

**Part 1: HW Review** *(5-10 min)*

- Ask participants to share what they wrote about for HW.
- What challenge or decision did they face this week?
- How did you apply your values to help handle it?
- What went well? What would you change in the future?

***~35 min left***

**Part 2: Making Plans Based in Values Activity** *(10 min)*

- Pass out “**7. Making My Plans for Now and the Future**” sheets
- Participants are asked to think about ways in which they can live more in accordance with their values. They should have prompts similar to the following:
  - Think about a long-term goal you have related to your values
  - Think about two things you’d like to do in a year in order to better live up to your values
  - Think about two things you can do in the next week to live more in accordance with you values

**Part 3: Discussion of Future Goals in the Short Term** *(10 min)*

- Lead a discussion among participants about their future goals with a focus now on their short-term goals for the next few weeks
- Sample questions:
  - What did you write for your long-term goals related to your values?
  - How do these goals connect to your values?
  - How do you plan to work toward those goals in the next week? How about in the next year?
  - How could you handle problems getting in the way of your plans and change your plans if you need to?
- During this discussion, try and make connections between participants’ values and goals.
- Try to especially validate small, reasonable, and achievable goals, and emphasize that small steps are very important. We should celebrate small steps as well as big ones!

***~15 min left***

**Part 4: Letter to Someone You Admire** *(10 min)*

- Pass out sheet “**8. Letter to Someone You Admire**”
- Write a letter to someone else who has values you admire and acts on those values.
  - It’s okay if they don’t know exactly what the person’s values are. Guessing based on how they act and what they say is just fine.
  - They should be specific about how the person acts on their values and why they admire that person.
- Emphasize that students can give this letter to the person they write about if they want.

**Part 5: Homework** *(5 min)*

- - Assignment: Try and accomplish the goal you set for yourself for the next week and write about how it goes.
  - We will think about some of these questions: Did you encounter any challenges? How did you go about overcoming them? What would you want to keep doing in the future and what do you want to change?

**Session 4:**

- **Required sheets:**
  - 9. Thinking Through Challenges with Values
- **Session overview:**
  - Part 1: HW Review *(5 min)*
  - Part 2: Hypothetical and Real Scenarios *(15 min)*
  - Part 3: Conclusion Discussion *(10 min)*
  - Part 4: Closing Group Share Reflection *(5-7 min)*
  - Part 5: Endpoint measures (including feedback) *(20-25 min)*

**Part 1: HW Review** *(5 min)*

- Ask participants to share what they wrote about for HW:
  - Did they accomplish their goals?
  - What challenges did they face?
  - What might they want to change next week about how they act according to their values?

***~60 min left***

**Part 2: Hypothetical and Real Scenarios** *(15 min)*

- Pass out the sheet “**9. Thinking Through Challenges with Values**”
- Ask the students to break into groups of two for discussions
  - They will discuss how values might be useful in a hypothetical scenario, and then in real scenarios faced by the participants.
  - Say: “Think about the values that are most important to you, and how those values would impact your behavior. There are no right or wrong answers, and you do not need to share your answers with the broader group”

**Part 3: Conclusion Discussion** *(10 min)*

- Lead a wrap-up discussion about values. This discussion does **not** have to focus on the most recent activity.
  - Sample questions:
    - How can you use the ideas we’ve learned about in your lives?
    - Are there any strategies you want to use this week?
    - How can the stuff we talked about help you in your academics?
    - How can the stuff we learned about help you in your relationships?
    - How can the stuff we learned about help you overcome challenges?
- **EMPHASIZE** how **you can think more about your values and use them to inform your actions and decisions**.
- Considering and acting on your values can lead to improvements in being happy, building good friendships and family relationships, and being successful in school and work.
- You can always work to be closer to what you value, even if your life and your actions/thoughts will never be perfect.

***~35 min left***

**Part 4: Closing Group Share Reflection** *(5-7 min)*

- Ask everyone to go around and each say one thing they will use in the future or that they learned from the groups as a whole, not just the last session.

**Part 5: Endpoint measures (including feedback)** *(20-25 min)*

- **Pass out the questionnaires**
- Tell students to take about 20 minutes to fill them out
- Remind students before they fill out the document:
  - Their responses will be kept private (no one except the study team will see them)
  - No one at the school will see their responses – the administration and teachers will not have access to them.
  - There are no right or wrong answers; they will not be graded
  - You should answer as honestly as possible.
- Answer any questions that students have.
- Explain that this will be the last part of the program.
- Tell your students that you enjoyed working with them and thank them for being good students.
- When the students are finished, collect their questionnaires.

----------------------------------------------------------------------------------------------------------------
